# 3,3′-diindolylmethane inhibits LPS-induced human chondrocytes apoptosis and extracellular matrix degradation by activating PI3K-Akt-mTOR-mediated autophagy

**DOI:** 10.3389/fphar.2022.999851

**Published:** 2022-11-10

**Authors:** Hao Tang, Kunpeng Qin, Anquan Wang, Shuang Li, Sheng Fang, Weilu Gao, Ming Lu, Wei Huang, Hui Zhang, Zongsheng Yin

**Affiliations:** ^1^ Department of Orthopedics, The First Affiliated Hospital of Anhui Medical University, Hefei, China; ^2^ The Key Laboratory of Microbiology and Parasitology of Anhui Province, The Key Laboratory of Zoonoses of High Institutions in Anhui, Anhui Medical University, Hefei, China; ^3^ Department of Orthopedics, The Second People’s Hospital of Hefei, Hefei, China; ^4^ Department of Orthopedics, The First Affiliated Hospital of USTC, Division of Life Sciences and Medicine, University of Science and Technology of China, Hefei, China

**Keywords:** 3, 3′-diindolylmethane, osteoarthritis, apoptosis, autophagy, chondrocyte

## Abstract

Osteoarthritis (OA) is a chronic degenerative joint disease characterized by articular cartilage destruction. The pathological mechanisms are complex; in particular, inflammation, autophagy, and apoptosis are often involved. 3,3-Diindolylmethane (DIM), a phytoconstituent extracted from cruciferous vegetables, has various effects such as anti-inflammatory, antioxidant and anti-apoptotic. However, the effects of DIM on osteoarthritic chondrocytes remain undetermined. In this study, we simulated a lipopolysaccharide (LPS)-induced osteoarthritis model in human primary chondrocytes. We found that LPS stimulation significantly inhibited autophagy, induced chondrocyte apoptosis and extracellular matrix (ECM) degradation, which could be ameliorated by DIM. DIM inhibited the expression of a disintegrin and metalloproteinase with thrombospondin motif 5 (ADAMTS-5), matrix metalloproteinase 13 (MMP13), cleaved caspase-3, Bax, and p62, and increased the expression level of collagen II, aggrecan, Bcl-2, light chain 3 Ⅱ (LC3 Ⅱ), and beclin-1. Mechanistic studies showed that DIM increased chondrocyte autophagy levels by inhibiting the activation of PI3K/AKT/mTOR pathway. In mice destabilization of the medial meniscus (DMM) model, immunohistochemical analysis showed that DIM inhibited the expression of p-PI3K and cleaved caspase-3, increased the expression of LC3 Ⅱ. Furthermore, DIM relieved joint cartilage degeneration. In conclusion, our findings demonstrate for the first time that DIM inhibits LPS-induced chondrocyte apoptosis and ECM degradation by regulating the PI3K/AKT/mTOR-autophagy axis and delays OA progression *in vivo*.

## Introduction

Osteoarthritis (OA) is a degenerative joint disease that affects older people worldwide, characterized by progressive destruction and inflammation of articular cartilage (Glyn-Jones et al., 2015). The incidence of OA is reported to be increasing year by year, and the condition is a major cause of disability in older adults. Contemporary treatment mainly improves symptoms, and there is no effective treatment to prevent the progression of OA (Maudens et al., 2018). Therefore, it is clinically important to develop more convenient and effective interventions to slow down progression of the disease (Goldring, 2006). Degenerative changes of articular cartilage are the most important pathological changes in OA, and articular cartilage consists of only chondrocytes and extracellular matrix (ECM). The main matrix components of the ECM are collagen II and aggrecan, and the main ECM catabolic enzymes are matrix metalloproteinases (MMPs) and a disintegrin and metalloproteinase with thrombospondin motifs (ADAMTS) (Cucchiarini et al., 2016). Under normal physiological conditions, chondrocytes maintain the balance of ECM synthesis and catabolism to ensure the structural and functional integrity of cartilage (Musumeci et al., 2011a).

Recently, an increasing number of studies have shown that OA has diverse causative factors and a complex pathogenesis and determined that the release of inflammatory mediators causes inhibition of cellular autophagy, resulting in increased apoptosis, which ultimately leads to an imbalance in ECM metabolism and accelerates OA development (Li et al., 2019). Previous study has reported that apoptosis is positively correlated with the severity of cartilage destruction and matrix depletion in human OA tissue specimens (Musumeci et al., 2015). Chondrocytes freshly isolated from human OA cartilage displayed morphological evidence of apoptosis, while those from normal donors did not have any apoptotic cell signatures. These findings suggest that OA chondrocytes exhibit distinct apoptotic propensities (Musumeci et al., 2011a; Musumeci et al., 2011b). In addition, when chondrocyte homeostasis is unbalanced, the release of inflammatory mediators and catabolic enzymes is accelerated, the destruction of ECM speeds up, and OA progression occurs (Zhao et al., 2020).

Autophagy is a mechanism and a dynamic cellular process whereby damaged cellular components (such as organelles and proteins) are wrapped by membrane structures to form autophagic vesicles, which then fuse with lysosomes, and whose contents are degraded and recycled to maintain normal cellular metabolism (He and Klionsky, 2009; Ravikumar et al., 2010; Polewska, 2012; Li et al., 2016). It has been reported that normal cartilage tissue expresses abundant LC3 II, Beclin1 and other important autophagy-related proteins, suggesting that autophagy may be involved in maintaining the normal physiological function and structural integrity of cartilage tissue (Sasaki et al., 2012; Zhang et al., 2015; Qin et al., 2017). In contrast, the expression of autophagy-related proteins is reduced in human OA chondrocytes (Loeser, 2011; Lian et al., 2018). Activation of autophagy increases the expression levels of both collagen II and aggrecan while reducing those of ADAMTS-5 and MMP-13 in chondrocytes and ameliorating arthritis progression (Qin et al., 2017; Huang et al., 2020; Lin et al., 2021). Furthermore, inhibition of autophagy in mice chondrocytes exacerbated arthritis progression (Bouderlique et al., 2016), while intra-articular injection of rapamycin to activate autophagy in a mice destabilization of the medial meniscus (DMM) model improved cartilage degeneration (Takayama et al., 2014).

3,3-diindolylmethane (DIM) is a natural product found in cruciferous edible plants, such as cauliflower, cabbage, and broccoli. Previous studies have reported that DIM has several preventive effects, especially displaying anti-tumor, anti-inflammatory, antioxidant and free radical scavenging roles (Luo et al., 2018; Ye et al., 2021). Recent studies have determined that DIM also protects against kidney, heart, and liver damage. It has been reported that DIM attenuated carbon tetrachloride–induced acute liver injury in mice by inhibiting inflammatory responses and apoptosis and by modulating oxidative stress (Munakarmi et al., 2020). DIM has been reported to attenuate oxidative stress–induced apoptosis in hippocampal neurons (Lee et al., 2019) and protect neuronal cells from inflammation and brain tissue ischemia (Rzemieniec et al., 2016; Lee et al., 2019). In addition, DIM attenuates lipopolysaccharide (LPS)-induced inflammatory responses and apoptosis in cardiomyopathy (Luo et al., 2018) and induces protective autophagy in prostate cancer (Draz et al., 2017). In a rat model of rheumatoid arthritis, DIM was observed to block osteoclast formation (Dong et al., 2010) and, through inhibition of the MAPK and AKT/mTOR pathways, inhibit synovial fibroblast proliferation, migration, invasion, and inflammatory factor release and attenuate experimental arthritis progression (Du et al., 2019).

Although previous studies have extensively investigated the function of DIM, the potential role of DIM in osteoarthritis and its effects on chondrocytes remain undetermined. In the present study, we observed the effects of DIM on LPS stimulation-induced chondrocyte autophagy, apoptosis, and ECM metabolism *in vitro* and explored the possible molecular mechanisms. The potential therapeutic function of DIM was also evaluated in a DMM mice model of OA.

## Materials and methods

### Experimental design

DIM was purchased from MCE (HY-15758, Monmouth Junction, NJ, United States) (purity >99%). LPS is currently the main pro-inflammatory inducer (Mayeux, 1997), which is commonly used to induce arthritis models in chondrocytes (Yoshino et al., 2000; Lorenz et al., 2013; Ding et al., 2019; Li et al., 2020; Zeng et al., 2021). So, we used LPS (10 µg/ml, Sigma-Aldrich) to treat human primary chondrocytes to simulate OA models. Six groups were established. The control group contained chondrocytes without any treatment. The negative control group contained chondrocytes to which only DIM (40 µM) was added. The LPS group contained chondrocytes only treated with LPS (10 ug/ml). The LPS + DIM group contained the chondrocytes treated with different concentrations of DIM (10, 20, 40 µM) for 2 h, and then LPS was added in the medium to stimulate chondrocytes for 24 h (without replacing the medium). We used 40 µM of DIM for the detection of immunofluorescence, flow cytometry, monodansylcadaverine (MDC) staining, transmission electron microscopy (TEM) and glycosaminoglycan (GAG) experiments.

*In vivo*, mice were randomly divided into a sham-operated group, an OA group (DMM), and a DIM-treated OA group (DMM + DIM) (*n* = 10 in each group). Mice DMM model creation took place in the OA group, and the DIM-treated OA group received a 50 mg/kg/day dose of DIM (*via* daily intraperitoneal injection for 8 weeks). Mice in the Sham and DMM groups received equal amounts of saline. All animals were executed 8 weeks after surgery, and cartilage samples were collected for immunological and histological analysis.

### Isolation and culture of primary chondrocytes

The collection of cartilage tissue involving human OA was approved by the Medical Ethics Committee of the First Affiliated Hospital of Anhui Medical University (ethics no. PJ2022-04-55), and all participants signed an informed consent form. Articular cartilage was obtained from patients who had undergone total knee arthroplasty at the First Affiliated Hospital of Anhui Medical University. The specimens were rapidly transferred to the operating table and washed 3–5 times with phosphate-buffered saline (PBS) in a sterile environment, and the cartilage tissue was then cut into bone pieces of 1 mm^3^ in size and then digested with 0.25% trypsin (Beyotime, Shanghai, China) for 30 min, followed by 0.2% collagenase Ⅱ (Sigma-Aldrich) in a 37°C incubator overnight. The digested chondrocytes were then resuspended and inoculated in culture flasks containing Dulbecco’s modified Eagle medium/nutrient mixture F-12, 10% fetal bovine serum, and 1% penicillin/streptomycin antibiotics; when 80%–90% fusion was achieved, the cells were then digested using 0.25% trypsin solution, centrifuged, resuspended, and passaged. The complete medium was changed every 2 days, and only cells of the first or second generation were used for the experiments to avoid phenotypic changes.

### Toluidine blue staining of chondrocytes

Chondrocytes were inoculated in 24-well plates and cultured for 24 h, then washed three times with PBS solution for 5 min/time and subsequently fixed in 4% paraformaldehyde solution for 20 min. After being washed with PBS, they were treated with 1% toluidine blue solution (Solarbio, Beijing, China) for 1 h at room temperature, washed with PBS again, dried and placed on slides, and then sealed with neutral gum. All chondrocytes were observed under a microscope (Tissue FAXS Plus S; Tissue Gnostics, Vienna, Austria) and photographed.

### Cell viability assay

The cytotoxicity of DIM on human chondrocytes was assayed with a cell counting kit 8 (CCK-8) (Beyotime). First, human chondrocytes were cultured in 96-well plates (5×10^3^ cells/well) for 24 h with different concentrations of DIM (0, 1, 5, 10, 20, 40, or 80 μM) for 24 or 48 h. After each well washed with PBS, 100 μL new medium containing 10% CCK8 was added to each well, incubated at 37°C for 2 h. The absorbance was then detected at 450 nm using a microplate reader (Leica Microsystems, Wetzlar, Germany).

### RNA extraction and quantitative real-time polymerase chain reaction (qRT-PCR)

The treated cells were washed with enzyme-free water, and total cellular RNA was subsequently extracted with TRIzol reagent (Invitrogen, Carlsbad, CA, United States), RNA purity was assessed according to a 260-/280-nm ratio using a NanoDrop One device (Thermo Fisher Scientific, Waltham, MA, United States), and RNA concentrations were measured using a reverse transcription kit (Takara Bio, Kusatsu, Japan) A 10-µL reaction system was set up to reverse-transcribe the extracted RNA to complementary DNA. Subsequently, qRT-PCR of the target genes was performed according to the SYBR^®^ Premix Ex Taq^™^ II kit (Takara Bio), and data analysis was performed with the Light Cycler 96 software (Roche, Alameda, CA, United States). The target gene messenger RNA levels were normalized to GAPDH levels, and the data obtained were analyzed using the 2^−ΔΔCT^ method. All experiments were repeated three times. The primer sequences were provided by Sangon (Shanghai, China) and are listed in Supplementary Table S1.

### Protein extraction and western blotting

A western blot technique was used to detect the expressions of related proteins. The treated cells were collected, radioimmunoprecipitation assay (RIPA) lysis buffer (Beyotime) and phenylmethylsulfonyl fluoride (PMSF) (Beyotime) were added, and the mixture was placed on ice for 30 min to lyse. Then, the supernatant was centrifuged and subsequently protein concentration was measured by BCA method (Beyotime) and added to 5 × sodium dodecyl sulfate–polyacrylamide gel electrophoresis (SDS-PAGE) protein loading buffer (Beyotime) and boiled for 10 min to obtain the total protein. Each well was loaded with 20ug protein, and then under the same GAPDH condition, the loading volume of each sample was calculated according to the required loading amount of protein and the sample protein concentration. The protein was subsequently separated by SDS-PAGE, then transferred to a polyvinylidene fluoride membrane (Millipore, Burlington, MA, United States) in constant flow. The membranes were subsequently closed with 5% skim milk for 2 h at room temperature. washed with tris-buffered saline and polysorbate 20 and incubated with the specific primary antibody overnight at 4°C, then washed and incubated with the corresponding specific secondary antibody for 2 h at room temperature, respectively. Finally, all band signals were detected using ECL ultrasensitive chemiluminescence reagents (Thermo Fisher Scientific, Waltham, MA, United States) and detected using the ImageJ version 1.53c software (U.S. National Institutes of Health, Bethesda, MD, United States) for quantitative analysis. The following antibodies were used in the western blot analysis: anti–collagen Ⅱ antibody (1:1000, 28459-1-AP, Proteintech Group, Rosemont, IL, United States), anti-aggrecan antibody (1:1000, 13880-1-AP, Proteintech Group), anti–ADAMTS-5 antibody (1:1000, Ab41037, Abcam, Cambridge, United Kingdom), anti–MMP-13 antibody (1:1000, 18165-1-AP, Proteintech Group), anti–LC3 A/B antibody (1:1000, 12741, CST, Danvers, MA, United States), anti-p62 antibody (1:10000, Ab109012, Abcam), anti–beclin-1 antibody (1:1000, Ab62557, Abcam), anti–cleaved caspase-3 antibody (1:1000, 5A1E, CST), anti-Bax antibody (1:5000, Ab32503, Abcam), anti–Bcl-2 antibody (1:1000, Ab32124, Abcam), anti–p-PI3K antibody (1:1000, ab151549, Abcam), anti–PI3K antibody (1:1000, 3358, CST), anti-AKT antibody (1:1000, 4691, CST), anti–phospho-AKT antibody (1:2000, 4060, CST), anti-mTOR antibody (1:5000, Ab32028, Abcam), anti–phospho-mTOR antibody (1:5000, Ab109268, Abcam), and anti-GAPDH antibody (1:10000, 60004-1-lg, Proteintech Group).

### Immunofluorescence staining

The treated cells were washed with PBS, then underwent 4% paraformaldehyde fixation for 20 min, were washed with PBS three more times, and underwent 0.3% Triton X-100 permeabilized for 15 min at room temperature. The cells were then closed with 10% bovine serum albumin (BSA) (goat serum blocking solution, Beyotime) for 1 h, then were washed with PBS and incubated overnight at 4°C in a wet box with the appropriate specific primary antibody, as follows: anti–collagen Ⅱ antibody (1:300, 28459-1-AP, Proteintech Group), anti–MMP-13 antibody (1: 300, 18165-1-AP, Proteintech Group), anti–LC3 A/B antibody (1:200, 12741, CST), anti–cleaved caspase-3 antibody (1:400, 5A1E, CST), or anti–p-PI3K antibody (1:300, Ab151549, Abcam). After three washes, the cells were incubated with fluorescein isothiocyanate (FITC) or rhodamine-labeled secondary antibody (1:100ZF-0311/ZF-0316, ZSGB-BIO, Beijing, China) for 1 h at room temperature and protected from light. Subsequently, 4′,6-diamidino-2-phenylindole (DAPI) staining solution (Beyotime) was added to label cell nuclei for 5 min, and finally anti-fluorescence quenching mounting solution was added dropwise. Images were observed and obtained under an automatic positive fluorescence microscope (DM6B; Leica, Wetzlar, Germany) and were quantified using ImageJ version 1.53c (U.S. National Institutes of Health, Bethesda).

### MDC staining

MDC is one of the most used fluorescent probes for cellular autophagy detection. It can specifically label autophagosomes through ion capture and specific binding to membrane lipids (Biederbick et al., 1995; Niemann et al., 2000; Niemann et al., 2001; Vázquez and Colombo, 2009). During our study, treated chondrocyte crawls were fixed in 4% paraformaldehyde for 20 min, washed three times with PBS, and incubated with MDC staining solution (Beyotime) for 60 min at 37°C in an incubator protected from light. Then, we washed them three times with assay buffer. Green fluorescence was observed under an automatic positive fluorescence microscope (DM6B; Leica).

### Transmission electron microscopy (TEM)

The treated cells were collected, then the samples were fixed with 2.5% glutaraldehyde in 0.1 M phosphate buffer (P885738, Macklin, Shanghai, China) at 4°C overnight. After washing with phosphate buffer, the samples were fixed in phosphate buffer with 1% OsO4 at 4°C for 2 h and rinsed thoroughly with ddH2O. The 2% aqueous uranyl acetate was used for *en bloc* staining for 2 h and then the samples were serially dehydrated with 50%, 70%, 90% and 100% alcohol and 100% acetone and embedded in epoxy resin for making the blocks of samples. Silver sections were cut with an ultramicrotome (EM UC7, Leica; thickness 70–90 nm), stained with lead citrate and uranyl acetate, and observed with an electron microscope (Talos L120C G2, Thermo Scientific, MA, United States).

### Cellular safranin O staining

Safranin O is a cationic dye that binds polyanions and binds to GAGs in chondrocytes to give them a red color, with the intensity of the red color being directly proportional to the GAG content. It is used to assess the content of GAGs in chondrocytes and can reflect the ability of chondrocytes to perform anabolic and catabolic activities. We washed the treated chondrocytes with PBS and fixed them with 4% paraformaldehyde for 20 min, then washed them with PBS and incubated them with safranin O staining solution (Solarbio, Beijing, China) for 30 min at room temperature. The cells were then washed with PBS and observed by microscopy (Tissue FAXS Plus S; Tissue Gnostics), and photographs were taken.

### Apoptosis analysis

The treated chondrocytes were collected in flow tubes, washed twice with cold PBS, and resuspended with 400 μL of 1 × annexin V conjugate using the annexin V-FITC/propidium iodide double-stained apoptosis assay kit (BestBio, Shanghai, China), followed by the addition of 5 μL of annexin V-FITC staining solution and incubation for 15 min at 4°C under light-proof conditions. Finally, 5 μL of propidium iodide (PI) staining solution was added and incubated for 3 min at 4°C under light-proof conditions, followed by flow cytometry (BD Celesta, San Jose, CA, United States). Flow analysis was performed with FlowJo version 10.6.0 (FlowJo LLC, Ashland, OR, United States).

### Animal model

Thirty 10-week-old C57BL/6 male wild-type mice were obtained from the Animal Center of Anhui Medical University. All surgical interventions, treatments, and postoperative animal care procedures were administered in accordance with the Guide for the Care and Use of Laboratory Animals of the National Institutes of Health and were performed in strict accordance with the requirements of the Animal Ethics Committee of Anhui Medical University (ethics no. LISC20190738). All the mice in the experiment were housed in standard experimental cages with a 12-h light/dark cycle and were freely access to water and standard food. Mice OA models were created by DMM surgery. Anesthesia was performed by intraperitoneal injection of an appropriate amount of 2% pentobarbital, followed by exposure of the joint capsule medial to the patellar tendon of the right knee and dissection of the medial meniscus tibial ligament with microsurgical scissors. In the sham group, only the joint capsule was incised, and the medial meniscal tibial ligament was not treated.

### Immunohistochemical assay

The obtained knee joints were fixed in 4% paraformaldehyde for 24 h, then decalcified, paraffin-embedded, cut into 5-μm sections, dewaxed, and rehydrated. Antigen repair was performed using the E enzyme method (DIG-3008; MXB, Fuzhou China), and appropriate amounts of rabbit- or mouse-derived endogenous peroxidase blocker (PV-6001/PV-6002, ZSGB-BIO) were added and incubated at room temperature for 10 min. After rinsing with PBS, an appropriate amount of primary antibody was added dropwise and incubated at 37°C for 60 min, using the appropriate specific primary antibody, as follows: anti–LC3 A/B antibody (1:300, 12741, CST), anti–cleaved caspase-3 antibody (1:1000, 5A1E, CST), anti-p-PI3K antibody (1:100, Ab151549, Abcam), anti–collagen Ⅱ antibody (1:800, 28459-1-AP, Proteintech Group), anti-aggrecan antibody (1:200, 13880-1-AP, Proteintech Group), anti–ADAMTS-5 antibody (1: 200, DF13268, Affinity), anti–MMP-13 antibody (1:200, 18165-1-AP, Proteintech Group). This was followed by the addition of the corresponding enzyme-labeled goat anti-rabbit or mouse immunoglobulin G polymer (PV-6001/PV-6002, ZSGB-BIO) dropwise to the section, then incubation at room temperature for 20 min; the addition of DAB (ZLI-9018, ZSGB-BIO) color development solution. Finally, re-staining, dehydration, transparency, and sealing of the section. The staining results were observed and interpreted by a qualified pathologist under a light microscope.

### Histopathological analysis

The obtained mice knee sections were dewaxed, hydrated, and stained with hematoxylin and eosin (H and E) staining as well as safranin O/fast green staining (Servicebio, Wuhan, China) to assess cartilage destruction. The extent of cartilage degeneration in the stained sections was assessed using the Osteoarthritis Research Society International (OARSI) scoring system (Glasson et al., 2010). The extent of synovial tissue changes in the stained sections was assessed using the Krenn Synovitis Score Criteria (Krenn et al., 2002; Krenn et al., 2006).

### X-ray imaging method

Eight weeks after DMM, mice were placed on a digital X-ray machine (Labscope, Glenbrook Technologies lnc, Randolph, NJ, United States) to undergo X-ray frontal and lateral imaging of all knee joints to assess the joint space, cartilage surface sclerosis, and bone formation. X-ray machine settings were 25 kV and 0.1 mA.

### Statistical analysis

All experiments were performed at least three times, and data are presented as mean ± standard deviation (SD) values. Statistical analysis was performed using the Prism version 9.0 software (GraphPad Software, San Diego, CA, United States). Statistically significant differences between the two groups were analyzed by *t* test, one-way ANOVA was used to compare multiple data groups. *p* < 0.05 was statistically significant.

## Results

### Identification of human chondrocytes

**FIGURE 1:**
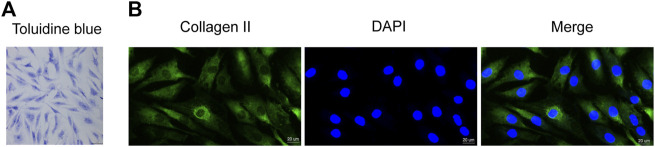
Identification of human primary chondrocytes and the effect of DIM on human OA chondrocyte viability **(A)** Proteoglycans in human primary chondrocytes were stained purple by toluidine blue (scale bar, 50 μm) **(B)** Collagen II immunofluorescence staining showed that the collagen II was stained green, while cell nuclei were stained blue by DAPI (scale bar, 20 μm).

First, we used toluidine blue staining and immunofluorescence staining to identify the isolated primary chondrocytes. Toluidine blue staining showed that the proteoglycans in the chondrocytes stained blue-purple, and the chondrocytes were spindle-shaped (Figure 1A). In addition, immunofluorescence staining showed greenish collagen II in the cytoplasm of chondrocytes and no positive staining in cell nuclei (Figure 1B). These two staining methods confirmed that the primary cells extracted from articular cartilage were chondrocytes.

### Effects of DIM on human chondrocyte viability

**FIGURE 2:**
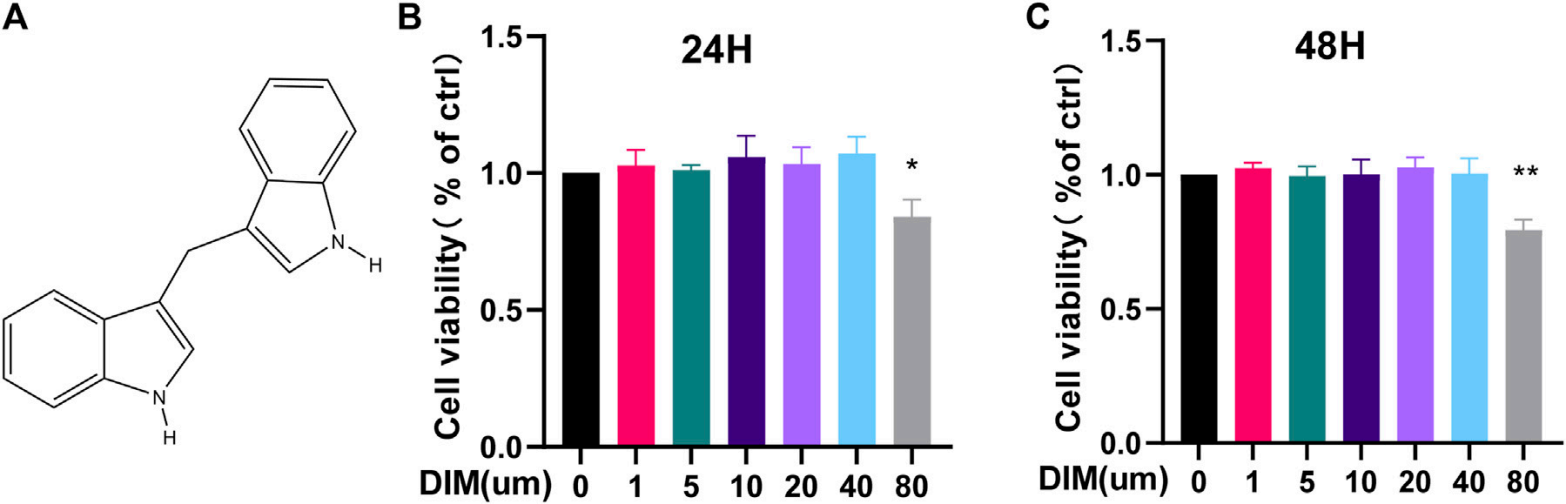
**(A)** Chemical structure of DIM **(B,C)** The cytotoxicity of DIM on chondrocytes was examined at various concentrations of DIM (0, 1,5, 10, 20, 40, and 80 μM) for 24 and 48 h by using the CCK-8 assay. The data are presented as mean ± SD values from 3 independent experiments. Using one-way ANOVA, * *p* <0.05, ** *p* <0.01 vs. the control.

The chemical structure of DIM is illustrated in Figure 2A. To clarify the cytotoxic effect of DIM on human chondrocytes, we assayed the cell viability by CCK-8 assay. Different concentrations of DIM (0, 1, 5, 10, 20, 40, and 80 μM) were incubated with chondrocytes for 24 and 48 h. After DIM treatment of chondrocytes for 24 and 48 h, there was no significant cytotoxic effect on primary chondrocytes at concentrations of 0–40 μM, but the high concentration of 80 μM reflected significant cytotoxicity and affected cell viability (Figures 2B,C). Therefore, the safe concentrations of 0, 10, 20, or 40 μM of DIM were used in subsequent related experiments.

### DIM alleviates LPS-induced ECM degradation in human chondrocytes

**FIGURE 3:**
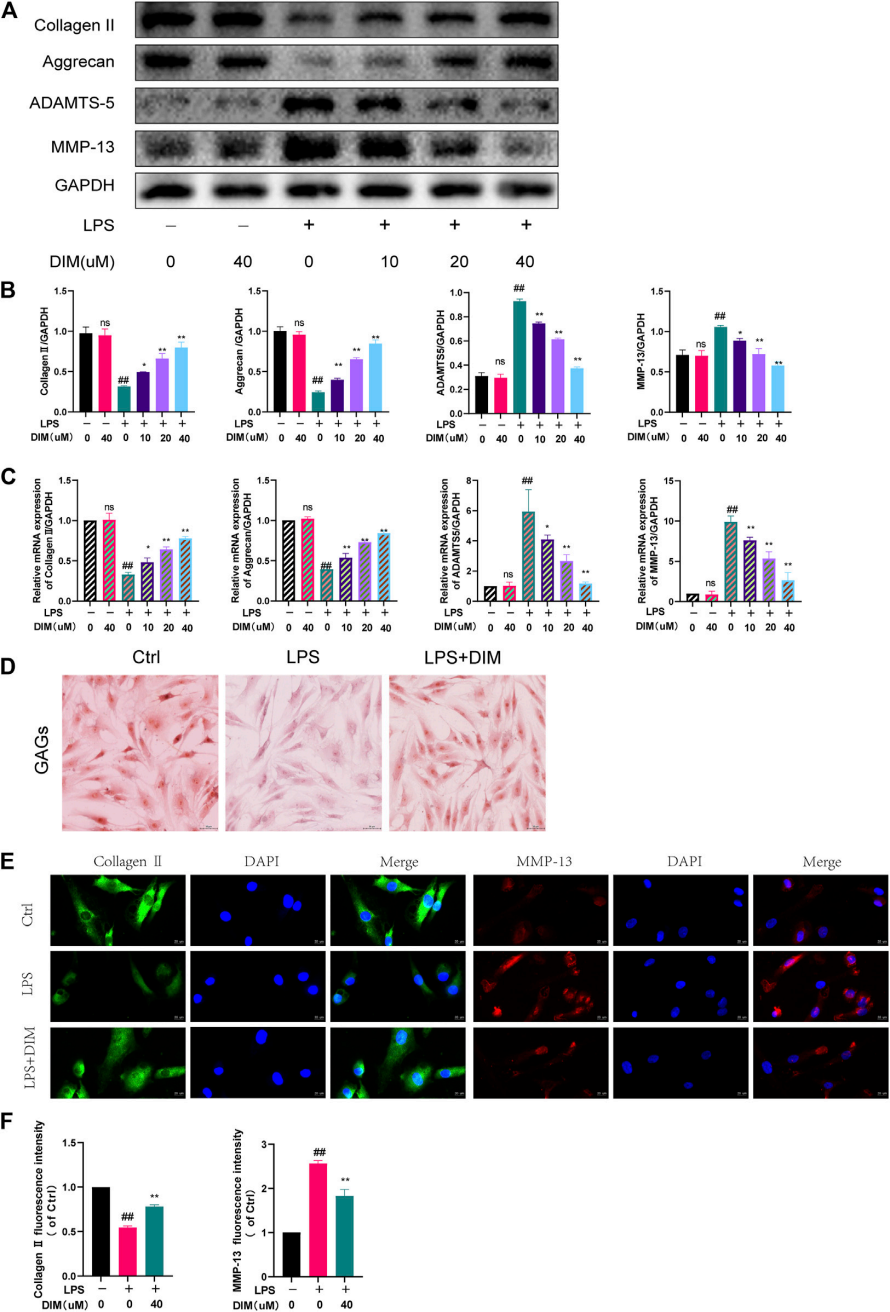
DIM alleviates LPS-induced ECM degradation in human chondrocytes **(A,B)** Effects of DIM on the protein expression levels of collagen II, aggrecan, ADAMTS-5, and MMP-13 in human chondrocytes treated as above were measured by western blotting and quantitation **(C)** Effects of DIM on the messenger RNA expression levels of collagen II, aggrecan, ADAMTS-5, and MMP-13 in human chondrocytes treated as above were measured by qRT-PCR **(D)** Safranin O staining of GAGs in human primary chondrocytes in each group (scale bar, 50 μm) **(E)** After treatment, immunofluorescence staining showed the changes in the fluorescence intensity of collagen II and MMP-13 in each group (scale bar, 20 µm) **(F)** The fluorescence intensity was measured with the ImageJ software (U.S. National Institutes of Health, Bethesda). The data are presented as mean ± SD values from three independent experiments. Using one-way ANOVA, NS, no statistical difference. ^#^ *p* <0.05 vs. the control; ^# #^ *p* <0.01 vs. the control; * *p* <0.05 vs. the LPS group; ** *p* < 0.01 vs. the LPS group.

We used western blot analysis (Figures 3A,B) and qRT-PCR (Figure 3C) to detect the expression of the following anabolic and catabolic indicators in cartilage: collagen II, aggrecan, ADAMTS-5, and MMP-13. Collagen II and aggrecan were significantly reduced in the LPS group, while ADAMTS-5 and MMP-13 were significantly increased. Pre-treatment with DIM reversed the downregulation of collagen II and aggrecan and the upregulation of ADAMTS-5 and MMP-13 induced by LPS stimulation in a dose-dependent relationship. We further examined the deposition of GAGs in human chondrocytes using safranin O staining, and GAG expression appeared significantly reduced in LPS-treated chondrocytes compared to controls, whereas DIM treatment ameliorated the LPS-induced loss of GAGs (Figure 3D, Supplementary Figure S2). In addition, we detected the expressions of collagen II and MMP-13 by immunofluorescence, and the results of fluorescence analysis were consistent with the western blot results (Figures 3E,F). Overall, the results showed that DIM ameliorated LPS-induced human primary chondrocytes anabolic and catabolic imbalance, which in turn reflected that DIM improved LPS-induced ECM degradation.

### DIM attenuates LPS-induced apoptosis in human chondrocytes

**FIGURE 4:**
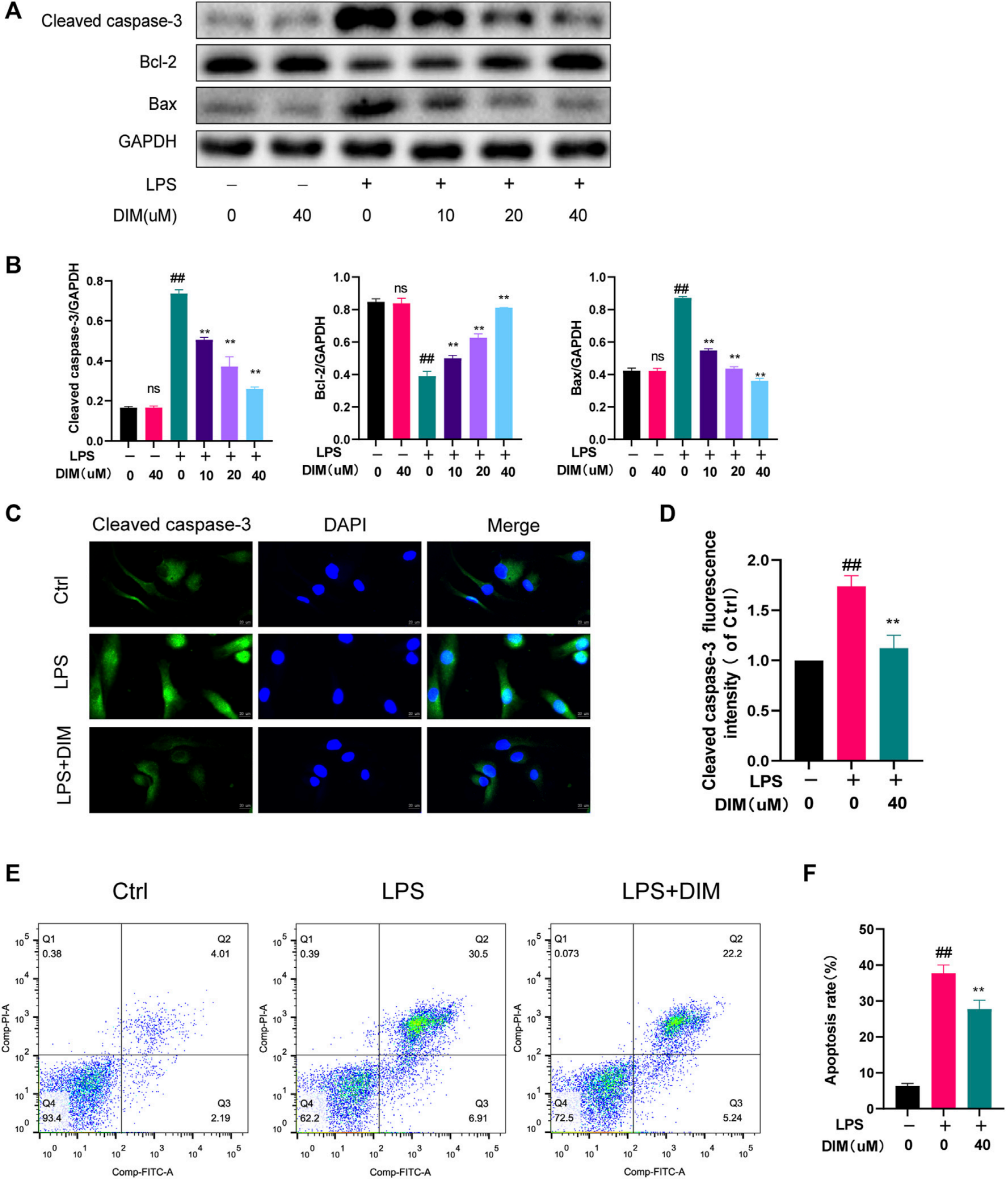
DIM attenuates LPS-induced apoptosis in human chondrocytes **(A,B)** Effects of DIM on the protein expression levels of cleaved caspase-3, Bcl-2, and Bax in human chondrocytes treated as above were measured by western blotting and quantitation **(C)** After treatment, immunofluorescence staining showed the changes in the fluorescence intensity of cleaved caspase-3 in each group (scale bar, 20 µm) **(D)** The fluorescence intensity was measured with the ImageJ software (U.S. National Institutes of Health, Bethesda) **(E,F)** After treatment, the results of flow cytometry detection of apoptotic cells in each group and quantitation. The data are presented as mean ± SD values from three independent experiments. Using one-way ANOVA, NS, no statistical difference. ^#^ *p* <0.05 vs. the control; ^# #^ *p* <0.01 vs. the control; * *p* <0.05 vs. the LPS group; ** *p* <0.01 vs. the LPS group.

We detected the expressions of Bax, Bcl-2, and cleaved caspase-3 by western blot analysis and further detected the expression of cleaved caspase-3 by immunofluorescence staining. Compared to the control group, LPS stimulation upregulated Bax and cleaved caspase-3 expression, while Bcl-2 expression was significantly down-regulated. In contrast, the expressions of Bax and cleaved caspase-3 were significantly down-regulated in the LPS group with the addition of DIM pre-treatment, while Bcl-2 expression was significantly increased (Figures 4A,B). Similarly, immunofluorescence staining for labeled cleaved caspase-3 results showed that DIM treatment decreased the intensity of cleaved caspase-3 (Figures 4C,D). In addition, apoptotic cells were examined by flow cytometry, and LPS caused an increase in chondrocyte apoptosis compared to the control group. However, the percentage of LPS-induced apoptosis was significantly reduced following pre-treatment with 40 μM of DIM (Figures 4E,F). These data suggest that DIM has an anti-apoptotic effect in human chondrocytes.

### DIM relieves the LPS-induced inhibition of autophagy and enhances autophagic flux in chondrocytes

**FIGURE 5:**
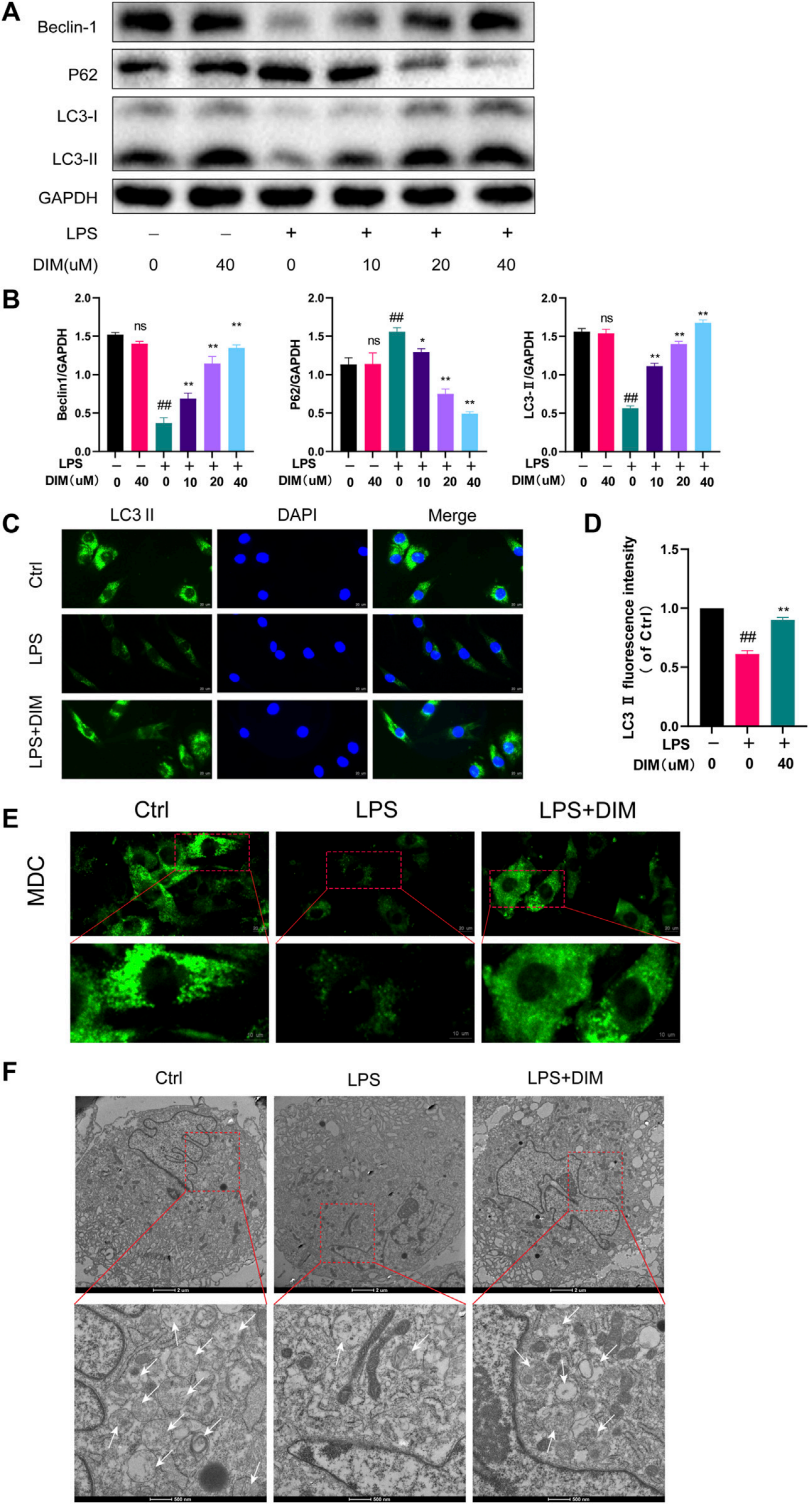
DIM relieves the LPS-induced inhibition of autophagy **(A,B)** Effects of DIM on the protein expression levels of beclin-1, LC3 II, and p62 in human chondrocytes treated as above were measured by western blotting and quantitation **(C)** After treatment, immunofluorescence staining showed the changes in the fluorescence intensity of LC3 II in each group (scale bar, 20 µm) **(D)** The fluorescence intensity was measured with the ImageJ software (U.S. National Institutes of Health, Bethesda) **(E)** After treatment, the effects of DIM and LPS on the number of chondrocytes autophagosomes were observed by MDC staining (punctate green staining represents autophagosomes; scale bar, 20 μm; partially enlarged image scale bar, 10 µm) **(F)** Microstructural detection of autophagosomes by transmission electron microscopy (White arrows indicate the formation of autophagosomes. Scale bar = 2 μm; partially enlarged image scale bar, 500 nm). The data are presented as mean ± SD values from three independent experiments. NS, no statistical difference; ^#^ *p* <0.05 vs. the control; ^# #^ *p* <0.01 vs. the control; * *p* <0.05 vs. the LPS group; ** *p* <0.01 vs. the LPS group.

Autophagy is a common mechanism for the removal of redundant or damaged organelles during development and aging. Moreover, autophagy plays a key role in regulating energy cycling and cellular homeostasis in chondrocytes (Mizushima and Levine, 2020). To investigate whether DIM activates autophagy in chondrocytes, we performed western blot analysis to assess changes in beclin-1, LC3 II, and p62 protein levels in human chondrocytes under different treatment conditions, which play a key role in the onset of autophagy (Glick et al., 2010; Boya et al., 2013). In LPS-treated primary human chondrocytes, a significant increase in LC3 II and beclin-1 levels and a significant decrease in p62 levels were observed in cells pre-treated with increased DIM (Figures 5A,B). Then, immunofluorescence staining results showed that DIM pre-treatment significantly increased the expression of LC3 II in LPS-stimulated chondrocytes (Figures 5C,D). In addition, MDC is an eosinophilic fluorescent dye that is specific for autophagosome formation. we assessed autophagic flux by MDC staining and transmission electron microscopy to observe the effect of DIM as well as that of LPS on the number of autophagosomes in chondrocytes; the intensity of MDC staining was diminished and the number of autophagosomes was reduced in LPS-treated chondrocytes compared to the blank control group. The intensity of MDC staining and the number of autophagosomes were significantly increased in primary human chondrocytes in the LPS + DIM group compared to the LPS group (Figures 5E,F). Based on these results, DIM could ameliorate the LPS-induced inhibition of autophagy and restore chondrocyte autophagy levels.

### DIM inhibits activation of the PI3K/AKT/mTOR signaling pathway

**FIGURE 6:**
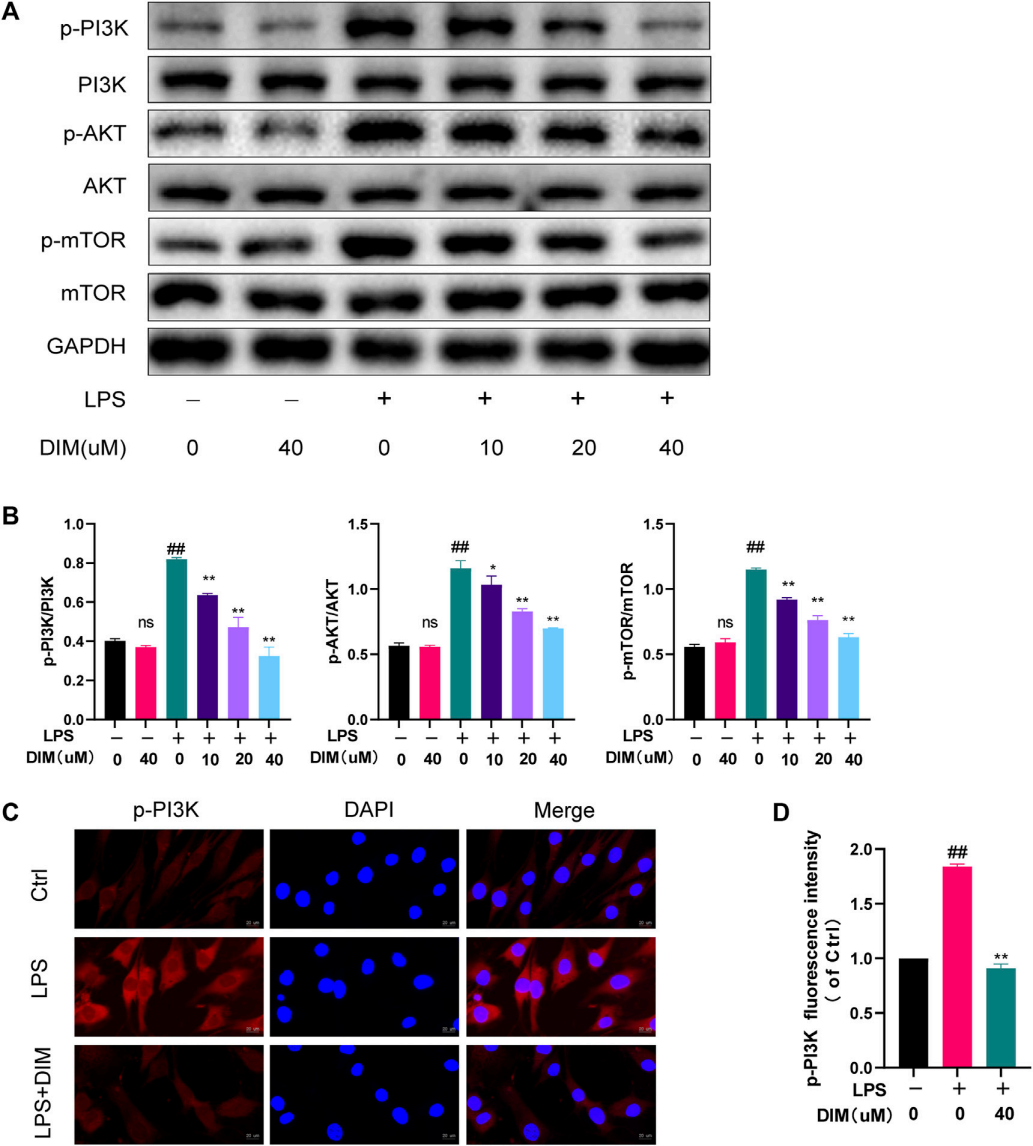
DIM inhibits the activation of the PI3K/AKT/mTOR signaling pathway **(A,B)** Effects of DIM on the protein expression levels of p-PI3K, total PI3K, p-AKT, total AKT, p-mTOR, and total mTOR in human chondrocytes treated as above were measured by western blotting and quantitation **(C)** After treatment, immunofluorescence staining showed the changes in the fluorescence intensity of p-PI3K in each group (scale bar, 20 µm) **(D)** The fluorescence intensity was measured with the ImageJ software (U.S. National Institutes of Health, Bethesda). The data are presented as mean ± SD values from three independent experiments. Using one-way ANOVA, NS, no statistical difference; ^#^ *p* <0.05 vs. the control; ^# #^ *p* <0.01 vs. the control; * *p* < 0.05 vs. the LPS group; ** *p* < 0.01 vs. the LPS group.

It is well known that the PI3K/AKT/mTOR signaling pathway is a key regulatory pathway for autophagy, and activation of the PI3K/AKT/mTOR pathway in OA is associated with autophagy inhibition (Xue et al., 2017). To confirm whether the mechanism of enhanced autophagy by DIM is related to the PI3K/AKT/mTOR signaling pathway, p-PI3K, p-AKT, and p-mTOR were detected by western blot analysis. The results showed that LPS treatment activated the PI3K/AKT/mTOR pathway compared to the control group, and the expressions of p-PI3K, p-AKT, and p-mTOR were significantly increased; meanwhile, the addition of DIM pre-treatment to the LPS group dose-dependently down-regulated the expressions of p-PI3K, p-AKT, and p-mTOR but did not affect the expressions of total PI3K, total AKT, or total mTOR (Figure 6A). The western blotting quantification analysis were based on the ratio of phosphorylated proteins (p-PI3K, p-AKT and p-mTOR) to total proteins (PI3K, AKT and mTOR) (Figure 6B). Immunofluorescence staining showed that DIM pre-treatment significantly inhibited p-PI3K activation (Figures 6C,D). These data suggest that the DIM can Inhibit activation of the PI3K/AKT/mTOR pathway in human chondrocytes.

### DIM enhances autophagy levels via the PI3K/AKT/mTOR signaling pathway

**FIGURE 7:**
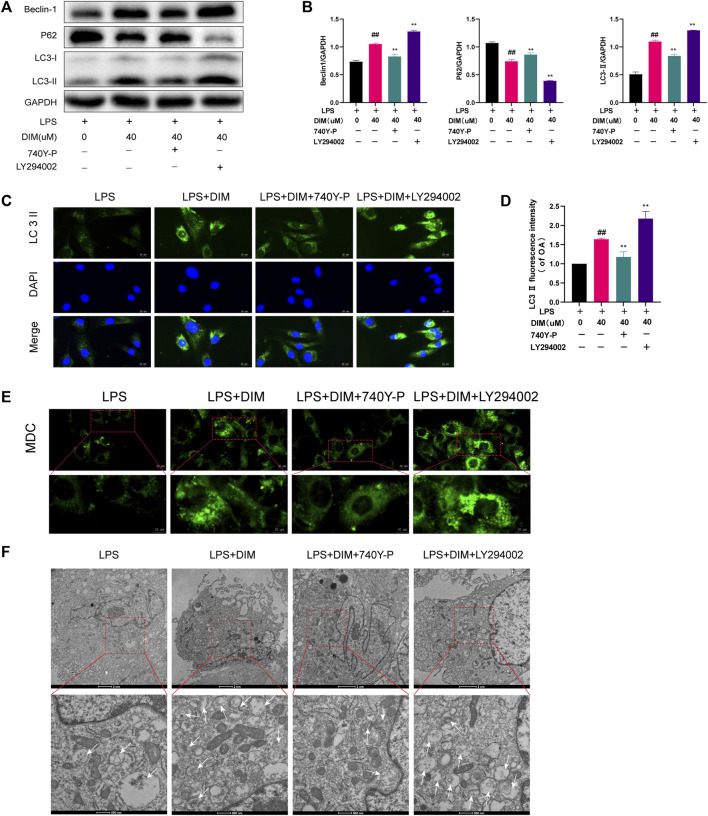
DIM enhances autophagy levels by modulating the PI3K/AKT/mTOR signaling pathway **(A,B)** Compared to the LPS + DIM group, the addition of PI3K activator (740Y-P) after treatment triggered a reduction in the level of autophagy, while the addition of PI3K inhibitor LY294002 increased the level of autophagy. Levels of autophagic marker proteins (beclin-1, p62, and LC3 II) were measured by western blotting and quantitation **(C)** The fluorescence intensity of LC3 II was detected by immunofluorescence staining (scale bar, 20 µm) **(D)** The fluorescence intensity was measured with the ImageJ software (U.S. National Institutes of Health, Bethesda) **(E)** The number of chondrocytes autophagosomes was observed by MDC staining (punctate green staining represents autophagosomes; scale bar, 20 μm; partially enlarged image scale bar, 10 µm) **(F)** Microstructural detection of autophagosomes by transmission electron microscopy (White arrows indicate the formation of autophagosomes. Scale bar = 2 µm; partially enlarged image scale bar, 500 nm). The data are presented as mean ± SD values from three independent experiments. Using one-way ANOVA, NS, no statistical difference; ^#^ *p* < 0.05 vs. the LPS group; ^# #^ *p* < 0.01 vs. the LPS group; * *p* < 0.05 vs. the LPS + DIM group; ** *p* < 0.01 vs. the LPS + DIM group.

To determine whether DIM-enhanced autophagy was mediated by the PI3K/AKT/mTOR pathway, the pathway activator 740Y-P (HY-P0175, MCE, Monmouth Junction, NJ, United States) and inhibitor LY294002 (HY-10108, MCE, Monmouth Junction, NJ, United States) were used for functional reversion experiments (Figures 7A,B). DIM increased LC3 II and beclin-1 expression levels and decreased p62 levels in the LPS group, but the increase in autophagy triggered by DIM was eliminated with 740Y-P, while the increase in autophagy triggered by DIM was enchanced with LY294002. Similar results were obtained for immunofluorescence labeling of LC3 II. Compared to the LPS + DIM group, the green fluorescence intensity of LC3 II decreased after 740Y-P use and increased after LY294002 use (Figures 7C,D). In addition, we observed the number of autophagosomes by MDC staining and transmission electron microscopy in chondrocytes. Compared to the LPS + DIM group, The intensity of MDC staining and the number of autophagosomes were significantly decreased after 740Y-P used and increased after LY294002 used (Figures 7E,F). These data suggested that the regulatory effect of DIM on autophagy was mediated *via* the PI3K/AKT/mTOR pathway.

### DIM attenuates LPS-induced apoptosis in chondrocytes *via* autophagy

**FIGURE 8:**
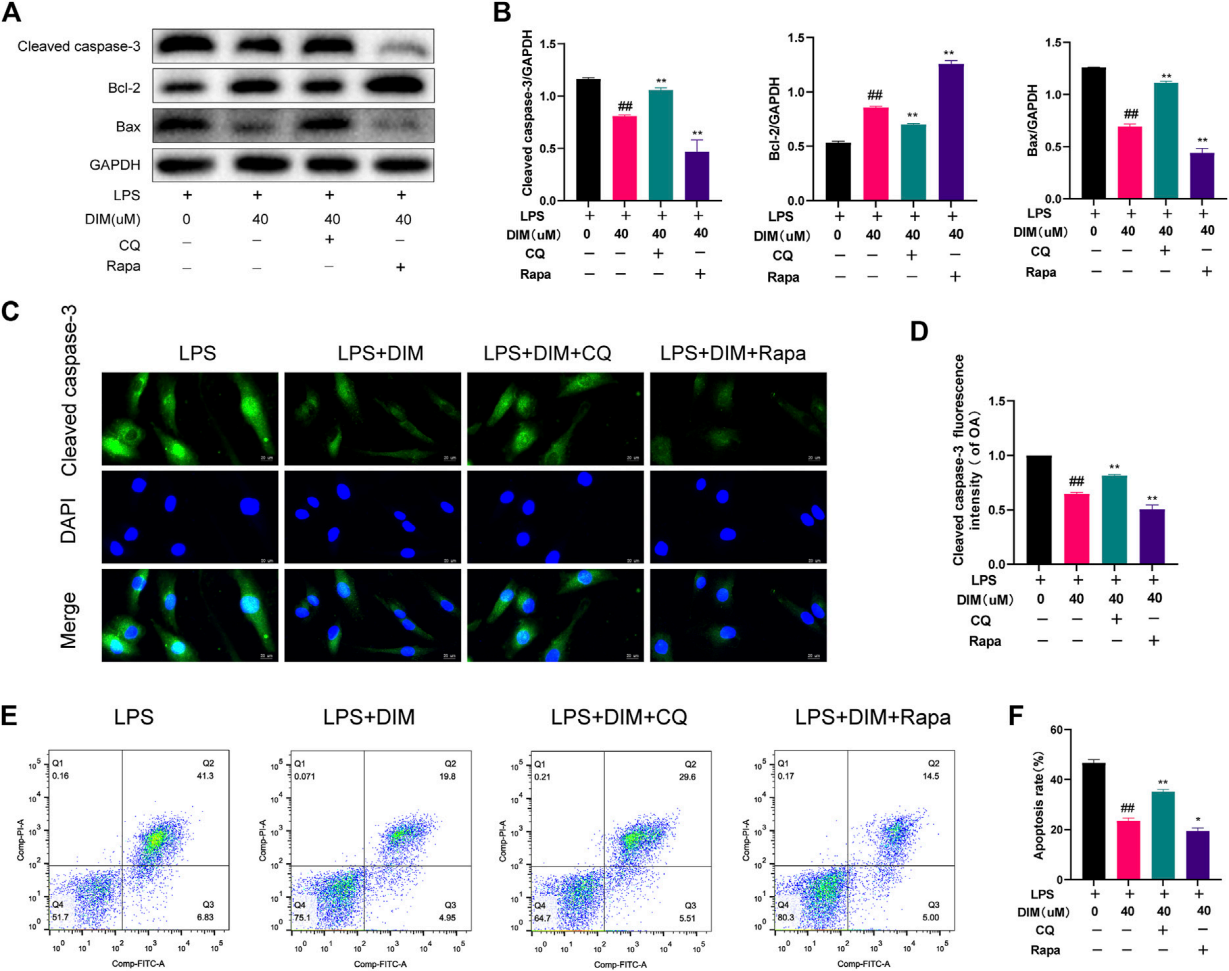
Activation of autophagy by DIM ameliorates LPS-induced chondrocyte apoptosis **(A,B)** Compared to the LPS + DIM group, the addition of an autophagy inhibitor (CQ) after treatment increased chondrocyte apoptosis, while the addition of an autophagy activator (Rapa) reduced chondrocyte apoptosis. Apoptosis marker proteins (cleaved caspase-3, Bcl-2, and Bax) were measured by western blotting and quantitation **(C)** The fluorescence intensity of cleaved caspase-3 was detected by immunofluorescence staining (scale bar, 20 µm) **(D)** the fluorescence intensity was measured with the ImageJ software (U.S. National Institutes of Health, Bethesda) **(E,F)** The apoptosis rate of chondrocytes was detected by flow cytometry and quantitation. The data are presented as mean ± SD values from three independent experiments. Using one-way ANOVA, NS, no statistical difference; ^#^ *p* <0.05 vs. the LPS group; ^# #^ *p* <0.01 vs. the LPS group; * *p* <0.05 vs. the LPS + DIM group; ** *p* <0.01 vs. the LPS + DIM group.

We used chloroquine (CQ), an autophagy inhibitor (HY-17589A, MCE), and rapamycin (Rapa), an autophagy activator (HY-10219, MCE), to determine whether autophagy was involved in the inhibitive effects of DIM on apoptosis. Compared to the LPS + DIM group, CQ effectively abolished the effects of DIM on reducing cleaved caspase-3 and Bax expression levels as well as increasing Bcl-2 expression levels. Compared to the LPS + DIM group, Rapa further reduced cleaved caspase-3 and Bax expression levels as well as increased Bcl-2 expression levels (Figures 8A,B). Additionally, we also detected the expression level of cleaved caspase-3 by immunofluorescence staining (Figures 8C,D) and the apoptosis of chondrocytes by flow cytometry (Figures 8E,F). Compared to the LPS + DIM group, the fluorescence intensity of cleaved caspase-3 and the chondrocyte apoptosis were significantly increased after CQ treatment. In contrast, both the fluorescence intensity of cleaved caspase-3 and chondrocyte apoptosis were reduced after Rapa treatment. These data suggested that inhibition of chondrocyte apoptosis by DIM in LPS-treated chondrocytes is mediated by autophagy.

### DIM alleviates OA cartilage degeneration in the surgical DMM mice model

**FIGURE 9:**
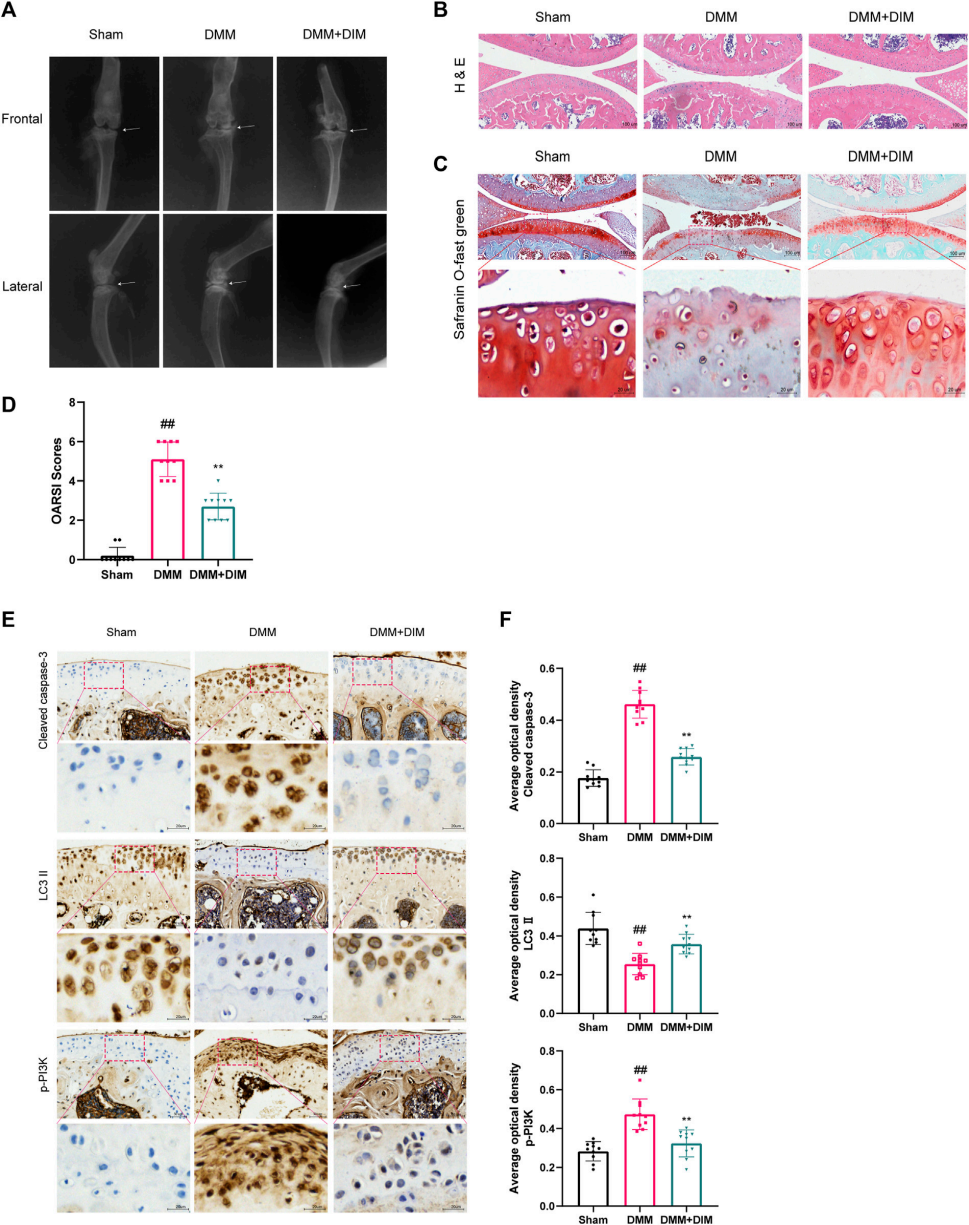
DIM alleviates OA development in the DMM mice model **(A)** Assessment of knee OA progression in a mice DMM model was conducted by digital X-ray imaging. Joint space narrowing was evident in the OA group (white arrows) **(B)** H and E staining of different experimental groups (scale bar, 100 µm) **(C)** Safranin O/fast green staining was used for the assessment of cartilage destruction (scale bar, 100 μm; partially enlarged image scale bar, 20 µm) **(D)** Mice articular cartilage OARSI scores **(E,F)** Immunohistochemical staining of cleaved caspase-3, LC3 II, and p-PI3K expressions in cartilage samples of different experimental groups (scale bar, 50 μm; partially enlarged image scale bar, 20 µm) and AODs were analyzed by the ImageJ software (U.S. National Institutes of Health, Bethesda). The data are presented as mean ± SD values from three independent experiments (n = 10 in each group). Using one-way ANOVA, NS, no statistical difference; ^#^ *p* < 0.05 vs. the Sham group; ^##^ *p* < 0.01, vs. the Sham group, * *p* < 0.05 vs. the DMM group; ** *p* < 0.01 vs. the DMM group.

We evaluated the therapeutic effect of DIM using a mice DMM model. The morphological and histological changes of the model mice were observed by H and E staining, safranin O/fast green staining, and X-ray analysis. X-ray (Figure 9A) and Hematoxylin and eosin staining (Figure 9B) results revealed that, compared to the Sham group, the joint space of the mice in the DMM group was significantly narrowed; the cartilage surface was hardened, rough, and uneven; and osteophyte counts were increased. However, DIM treatment alleviated this pathological manifestation. In addition, safranin O/fast green staining showed that, compared to the Sham group, the DMM group had significant loss of proteoglycan in the articular cartilage and aggravated cartilage erosion; however, these changes were improved after DIM treatment (Figure 9C). The OARSI scores and Synovitis Scores were consistent with the above pathological results, indicating that the DMM mice model treated with DIM were significantly different from the OA group, and the OARSI scores and Synovitis Scores of the DMM + DIM group were significantly lower than those of the OA group (Figure 9D, Supplementary Figure S3). Immunohistochemical results showed that the average optical densities (AODs) of p-PI3K and cleaved caspase-3 in the DMM group were significantly higher than those in the sham group, while the AOD of LC3 II was lower than that in the sham group. Compared to the DMM group, the AOD of LC3 II was significantly increased after DIM treatment, while the AODs of p-PI3K and cleaved caspase-3 were significantly decreased (Figures 9E,F). The AODs of MMP-13 and ADAMTS-5 in the DMM group were significantly higher than those in the sham group. Compared to the DMM group, the AODs of MMP-13 and ADAMTS-5 were significantly decreased after DIM treatment (Supplementary Figure S4). In addition, immunohistochemical staining analysis of Collagen II and Aggrecan showed that the AODs of Collagen II and Aggrecan in articular cartilage were lower in the DMM group than those in the sham group. Compared to the DMM group, the AODs of Collagen II and Aggrecan were significantly increased after DIM treatment (Supplementary Figure S5), consistent with the *in vitro* results.

## Discussion

OA is the most common joint disease and the leading cause of disability worldwide. It can limit the patient’s daily activities, such as walking and running and other dependent behaviors, and severely affecting their quality of life, causing a significant social burden, with a high prevalence in the elderly population. Recent epidemiological findings show that the prevalence of OA is 9.6% in elder men and 18% in elder women (Woolf and Pfleger, 2003; Verlaan et al., 2018). The most affected site of OA is the knee joint (Fransen et al., 2011). OA is caused by a variety of factors and is an extremely complex pathogenic process that involves several different pathophysiological mechanisms, including increased inflammatory stimulation, an imbalance in chondrocyte metabolism, increased apoptosis, and degradation of the cartilage matrix (Li et al., 2019). Current treatments are limited to oral medications, which can relieve joint swelling and pain but cannot completely cure the development of OA, and joint replacement surgery is still required in the long term, so there is still a need to develop safer and more effective drugs to treat OA (Vaishya et al., 2016).

DIM is a natural Fat-soluble small molecule compound harvested from cruciferous vegetables which belongs to the class of indole glucosinolate (Maruthanila et al., 2014; Shi et al., 2017). Due to its lipophilic nature, DIM can directly cross the cell membrane into the cytoplasm to exert biological activity (Jellinck et al., 1993; Carpenter et al., 2016; Khan et al., 2019; Ong and Amstad, 2019; Martinotti et al., 2020). DIM can intracellularly regulate a variety of signaling pathways as well as signaling enzymes (Laiakis et al., 2020). For example, DIM can block PI3K/Akt/MTOR/NF-κB signaling (Ahmad et al., 2013), activate AMP-activated protein kinases (Chen et al., 2012), inhibit nuclear factor kappa B (Cho et al., 2008; Weng et al., 2012), inhibit cell cycle protein-dependent kinases (Kim et al., 2012), and reduce androgen receptor levels (Palomera-Sanchez et al., 2017). In recent years, an increasing number of studies have explored the chondroprotective effects of plant components (Sukhikh et al., 2021). DIM has been found to have various health-promoting benefits. However, it remains unclear whether DIM has any effect on OA chondrocytes, which is explored in depth in this paper. We investigated the role and mechanism of DIM in arthritis both *in vivo* and *in vitro*.

It has been reported that chondrocytes are the only cell type that constitutes articular cartilage and are responsible for the synthesis and secretion of cartilage ECM macromolecules, such as collagen II and aggrecan (Goldring and Marcu, 2009; Musumeci et al., 2015; Guilak et al., 2018). In addition, chondrocytes also can synthesize matrix degrading enzymes, such as MMP-13 and ADAMTS-5 (Vincenti and Brinckerhoff, 2002; Rowan et al., 2008; Takahata et al., 2019; Meng et al., 2020). Currently, the synthesis and degradation status of cartilage matrix is mainly reflected indirectly by detecting anabolic and catabolic marker proteins as well as proteoglycan moieties in chondrocytes (Chang et al., 2019; Huang et al., 2020; Lin et al., 2021). Under normal physiological conditions, chondrocytes maintain a balance between the synthesis and degradation of ECM components to ensure the structural and functional integrity of cartilage (Musumeci et al., 2011a). However, in the pathogenesis of OA, chondrocytes produce excess matrix-degrading enzymes to damage the extracellular matrix, particularly MMP-13 and ADAMTS-5 (Kapoor et al., 2011). MMP-13 is a subclass of collagenase with the function of cleaving ECM collagen II (Bramono et al., 2004), while ADAMTS-5 is a zinc protein hydrolase that destroys aggrecan (Gendron et al., 2007). The decrease in matrix component synthesis and the increase in chondrocyte matrix breakdown by catabolic proteins resulted in loss of cartilage matrix components and increased cartilage destruction. It has been reported that therapeutic substances targeting MMP-13 and ADAMTS-5 may be ideal agents for the treatment of OA (Burrage et al., 2006; Mead and Apte, 2018). In the present study, we found that DIM acts against LPS-induced chondrocyte matrix degradation with a protective effect, and DIM significantly inhibited the expression of catabolic indicators MMP-13 and ADAMTS-5 and increased the expression of anabolic indicators collagen II and aggrecan in cartilage ECM.

Increased apoptosis is another major cause of matrix degradation. Apoptosis is a form of programmed cell death that can be activated through several different pathways, including death receptor–mediated and mitochondria-dependent apoptosis (Musumeci et al., 2011a; Zamli and Sharif, 2011). Among the biomarkers of OA, inflammatory mediators play a clear role in chondrocyte apoptosis. The synthesis of pro-inflammatory cytokines, which contribute to increased apoptosis, is involved in the degeneration of the cartilage ECM (Wu et al., 2007; Musumeci et al., 2011b). Indeed, in human OA tissue specimens, dissolution and calcification of the descending ECM correlate with apoptosis, and the rate of apoptosis is positively correlated with the severity of OA (Musumeci et al., 2011a). Abnormal mechanical stress on normal cartilage can lead to chondrocyte apoptosis and result in cartilage degeneration and loss (Kim et al., 2002). In addition, the literature reports that DIM exerts neuroprotective effects through the production of brain-derived neurotrophic factor and antioxidant enzymes in oxidative stress–induced apoptosis in hippocampal neurons (Lee et al., 2019). We found that chondrocyte apoptosis was significantly increased after LPS stimulation, while pre-treatment with the addition of DIM reversed LPS-induced chondrocyte apoptosis and revealed an increase in anti-apoptotic protein levels (Bcl-2) and a decrease in pro-apoptotic protein levels (Bax and cleaved caspase-3). This indicates that DIM can also play an anti-apoptotic role in chondrocytes.

Our previous studies have demonstrated that ECM degradation can be improved by activating autophagy in OA chondrocytes (Huang et al., 2020; Wang et al., 2022). Next, we conducted an in-depth study of the mechanism by which DIM inhibits LPS-induced chondrocyte apoptosis *in vitro*. Previously, it was reported that, in human prostate cancer cells, DIM induced cytoprotective autophagy through the induction of AMPK activation by AEG-1 (Draz et al., 2017). DIM inhibited the proliferation of gastric cancer cells through the miR-30e/ATG5 pathway involved in autophagy control (Ye et al., 2016). Therefore, we hypothesized that the molecular mechanism by which DIM exerts its protective effect may be related to restoring the level of chondrocyte autophagy. Cellular autophagy is an important protective mechanism for cells to maintain homeostasis and survival in the internal environment (Mizushima, 2007; He and Klionsky, 2009; Ryter et al., 2013), and changes in inflammation, starvation, pathogen infection, and endoplasmic reticulum stress can cause alterations in autophagy. In addition, autophagy is known to regulate a variety of cellular processes, such as apoptosis, pathogen removal, antigen presentation, and inflammation and is associated with many human diseases (Levine and Kroemer, 2008; Doria et al., 2013). Autophagy has been widely reported to be associated with the development of OA, and autophagic flux has been suggested as a possible therapeutic target for OA (He and Cheng, 2018; Huang et al., 2020; Xu et al., 2021). *In vitro*, we found that autophagy in chondrocytes after LPS stimulation was significantly decreased and LC3 II and beclin-1 protein expression levels were decreased, p62 protein expression was increased, the number of autophagic vesicles was decreased, and autophagic flux was blocked, but these phenomena were significantly reversed after the application of DIM treatment. We employed CQ and Rapa, as a specific inhibitor and activators of autophagy, to determine whether DIM regulates apoptosis through autophagy. We found that DIM ameliorated LPS-induced apoptosis, but CQ reversed this phenomenon; while activation of autophagy by Rapa further inhibited apoptosis, acting synergistically with DIM. Our results indicate that DIM inhibits LPS-induced chondrocyte apoptosis by restoring the level of chondrocyte autophagy.

The literature reports that the PI3K/AKT/mTOR signaling pathway regulates a variety of cellular processes, including cellular autophagy, metabolism, inflammation, metabolism, angiogenesis, and the cell cycle (Malemud, 2015). It is also an important regulator of chondrocyte autophagic flux, and inhibition of PI3K/AKT/mTOR signaling pathway activation to promote increased autophagy is an effective strategy to improve OA symptoms (Xue et al., 2017; He and Cheng, 2018; Han et al., 2021). In our study, we found that the PI3K/AKT/mTOR signaling pathway was activated after LPS stimulation of chondrocytes compared to the normal group, and the expressions of p-PI3K, p-AKT, and p-mTOR were increased, while DIM treatment significantly inhibited LPS-induced activation of this pathway. Moreover, we employed the PI3K agonist 740Y-P and PI3K inhibitor LY294002 to determine whether DIM regulates autophagy through PI3K/AKT/mTOR signal pathway. We found that activation of the PI3K/AKT/mTOR pathway by 740Y-P reversed DIM-induced autophagy recovery, while LY294002 inhibited the activation of the PI3K/AKT/mTOR pathway and further enhanced autophagy in chondrocytes, synergizing with DIM. The results showed that DIM enhanced the autophagy level of chondrocytes by inhibiting the PI3K/AKT/mTOR signaling pathway.

*In vivo*, we used the DMM method to establish a C57BL/6 mice OA model, which was assessed by hematoxylin and eosin staining, X-ray, safranin O/fast green staining, and immunohistochemistry. We found that, compared to mice in the sham group, the model mice in the DMM group showed severe cartilage destruction in the knee joint with rough surfaces, massive proteoglycan loss, reduced autophagy protein levels, increased apoptotic protein levels, and activated pathway proteins, while DIM treatment improved these symptoms, reversed chondrocyte apoptosis and ECM degradation, and reduced the OARSI score in DMM mice. The results of *in vivo* and *in vitro* were generally consistent. Taken together, the present study showed that DIM, as an easily accessible botanical component, not only improved the degeneration of articular cartilage in mice, but also had a significant protective effect on inflammation-induced chondrocyte destruction. Therefore, it is of great value to further investigate the potential applications of DIM in the treatment of osteoarthritis.

## Conclusion

**FIGURE 10:**
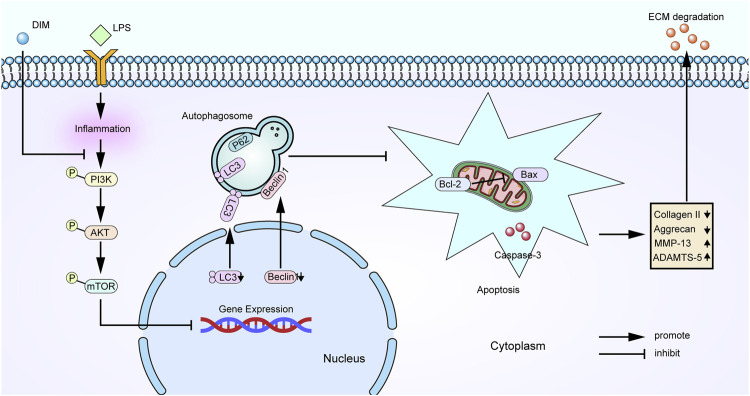
Schematic representation of the role of DIM in OA chondrocytes.

We found that DIM can act as a protective agent for chondrocytes. More importantly, our results reveal the mechanism by which DIM exerts its chondroprotective effects (Figure 10). DIM can reduce inflammation-induced chondrocytes apoptosis and extracellular matrix degradation by activating PI3K/AKT/mTOR-mediated autophagy. This study provides new guiding directions for DIM as a promising drug for the treatment of OA.

## Data Availability

The original contributions presented in the study are included in the article/Supplementary Material, further inquiries can be directed to the corresponding authors.

## References

[B1] Ahmad A. Biersack B. Li Y. Kong D. Bao B. Schobert R. (2013). Targeted regulation of PI3K/Akt/mTOR/NF-κB signaling by indole compounds and their derivatives: Mechanistic details and biological implications for cancer therapy. Anticancer. Agents Med. Chem. 13 (7), 1002–1013. 10.2174/18715206113139990078 23272910 PMC3901097

[B2] Biederbick A. Kern H. F. Elsässer H. P. (1995). Monodansylcadaverine (MDC) is a specific *in vivo* marker for autophagic vacuoles. Eur. J. Cell Biol. 66 (1), 3–14. 7750517

[B3] Bouderlique T. Vuppalapati K. K. Newton P. T. Li L. Barenius B. Chagin A. S. (2016). Targeted deletion of Atg5 in chondrocytes promotes age-related osteoarthritis. Ann. Rheum. Dis. 75 (3), 627–631. 10.1136/annrheumdis-2015-207742 26438374 PMC4789686

[B4] Boya P. Reggiori F. Codogno P. (2013). Emerging regulation and functions of autophagy. Nat. Cell Biol. 15 (7), 713–720. 10.1038/ncb2788 23817233 PMC7097732

[B5] Bramono D. S. Richmond J. C. Weitzel P. P. Kaplan D. L. Altman G. H. (2004). Matrix metalloproteinases and their clinical applications in orthopaedics. Clin. Orthop. Relat. Res. 428, 272–285. 10.1097/01.blo.0000144166.66737.3a 15534553

[B6] Burrage P. S. Mix K. S. Brinckerhoff C. E. (2006). Matrix metalloproteinases: Role in arthritis. Front. Biosci. 11, 529–543. 10.2741/1817 16146751

[B7] Carpenter T. S. Parkin J. Khalid S. (2016). The free energy of small solute permeation through the *Escherichia coli* outer membrane has a distinctly asymmetric profile. J. Phys. Chem. Lett. 7 (17), 3446–3451. 10.1021/acs.jpclett.6b01399 27518381

[B8] Chang S. H. Mori D. Kobayashi H. Mori Y. Nakamoto H. Okada K. (2019). Excessive mechanical loading promotes osteoarthritis through the gremlin-1-NF-κB pathway. Nat. Commun. 10 (1), 1442. 10.1038/s41467-019-09491-5 30926814 PMC6441020

[B9] Chen D. Banerjee S. Cui Q. C. Kong D. Sarkar F. H. Dou Q. P. (2012). Activation of AMP-activated protein kinase by 3, 3'-Diindolylmethane (DIM) is associated with human prostate cancer cell death *in vitro* and *in vivo* . PLoS One 7 (10), e47186. 10.1371/journal.pone.0047186 23056607 PMC3467201

[B10] Cho H. J. Seon M. R. Lee Y. M. Kim J. Kim J. K. Kim S. G. (2008). 3, 3'-Diindolylmethane suppresses the inflammatory response to lipopolysaccharide in murine macrophages. J. Nutr. 138 (1), 17–23. 10.1093/jn/138.1.17 18156398

[B11] Cucchiarini M. de Girolamo L. Filardo G. Oliveira J. M. Orth P. Pape D. (2016). Basic science of osteoarthritis. J. Exp. Orthop. 3 (1), 22. 10.1186/s40634-016-0060-6 27624438 PMC5021646

[B12] Ding Y. Wang L. Zhao Q. Wu Z. Kong L. (2019). MicroRNA-93 inhibits chondrocyte apoptosis and inflammation in osteoarthritis by targeting the TLR4/NF-κB signaling pathway. Int. J. Mol. Med. 43 (2), 779–790. 10.3892/ijmm.2018.4033 30569118 PMC6317687

[B13] Dong L. Xia S. Gao F. Zhang D. Chen J. Zhang J. (2010). 3, 3'-Diindolylmethane attenuates experimental arthritis and osteoclastogenesis. Biochem. Pharmacol. 79 (5), 715–721. 10.1016/j.bcp.2009.10.010 19854159

[B14] Doria A. Gatto M. Punzi L. (2013). Autophagy in human health and disease. N. Engl. J. Med. 368 (19), 1845–1846. 10.1056/NEJMc1303158 23656659

[B15] Draz H. Goldberg A. A. Titorenko V. I. Tomlinson Guns E. S. Safe S. H. Sanderson J. T. (2017). Diindolylmethane and its halogenated derivatives induce protective autophagy in human prostate cancer cells via induction of the oncogenic protein AEG-1 and activation of AMP-activated protein kinase (AMPK). Cell. Signal. 40, 172–182. 10.1016/j.cellsig.2017.09.006 28923415

[B16] Du H. Zhang X. Zeng Y. Huang X. Chen H. Wang S. (2019). A novel phytochemical, DIM, inhibits proliferation, migration, invasion and TNF-alpha induced inflammatory cytokine production of synovial fibroblasts from rheumatoid arthritis patients by targeting MAPK and AKT/mTOR signal pathway. Front. Immunol. 10, 1620. 10.3389/fimmu.2019.01620 31396207 PMC6663984

[B17] Fransen M. Bridgett L. March L. Hoy D. Penserga E. Brooks P. (2011). The epidemiology of osteoarthritis in Asia. Int. J. Rheum. Dis. 14 (2), 113–121. 10.1111/j.1756-185X.2011.01608.x 21518309

[B18] Gendron C. Kashiwagi M. Lim N. H. Enghild J. J. Thøgersen I. B. Hughes C. (2007). Proteolytic activities of human ADAMTS-5: Comparative studies with ADAMTS-4. J. Biol. Chem. 282 (25), 18294–18306. 10.1074/jbc.M701523200 17430884

[B19] Glasson S. S. Chambers M. G. Van Den Berg W. B. Little C. B. (2010). The OARSI histopathology initiative - recommendations for histological assessments of osteoarthritis in the mouse. Osteoarthr. Cartil. 18 (3), S17–S23. 10.1016/j.joca.2010.05.025 20864019

[B20] Glick D. Barth S. Macleod K. F. (2010). Autophagy: Cellular and molecular mechanisms. J. Pathol. 221 (1), 3–12. 10.1002/path.2697 20225336 PMC2990190

[B21] Glyn-Jones S. Palmer A. J. R. Agricola R. Price A. J. Vincent T. L. Weinans H. (2015). Osteoarthritis. Lancet 386 (9991), 376–387. 10.1016/s0140-6736(14)60802-3 25748615

[B22] Goldring M. B. Marcu K. B. (2009). Cartilage homeostasis in health and rheumatic diseases. Arthritis Res. Ther. 11 (3), 224. 10.1186/ar2592 19519926 PMC2714092

[B23] Goldring M. B. (2006). Update on the biology of the chondrocyte and new approaches to treating cartilage diseases. Best. Pract. Res. Clin. Rheumatol. 20 (5), 1003–1025. 10.1016/j.berh.2006.06.003 16980220

[B24] Guilak F. Nims R. J. Dicks A. Wu C. L. Meulenbelt I. (2018). Osteoarthritis as a disease of the cartilage pericellular matrix. Matrix Biol. 71-72, 40–50. 10.1016/j.matbio.2018.05.008 29800616 PMC6146061

[B25] Han G. Zhang Y. Li H. (2021). The combination treatment of curcumin and probucol protects chondrocytes from TNF-alpha induced inflammation by enhancing autophagy and reducing apoptosis via the PI3K-Akt-mTOR pathway. Oxid. Med. Cell. Longev. 2021, 5558066. 10.1155/2021/5558066 34257809 PMC8249126

[B26] He C. Klionsky D. J. (2009). Regulation mechanisms and signaling pathways of autophagy. Annu. Rev. Genet. 43, 67–93. 10.1146/annurev-genet-102808-114910 19653858 PMC2831538

[B27] He W. Cheng Y. (2018). Inhibition of miR-20 promotes proliferation and autophagy in articular chondrocytes by PI3K/AKT/mTOR signaling pathway. Biomed. Pharmacother. 97, 607–615. 10.1016/j.biopha.2017.10.152 29101804

[B28] Huang W. Cheng C. Shan W. S. Ding Z. F. Liu F. E. Lu W. (2020). Knockdown of SGK1 alleviates the IL-1β-induced chondrocyte anabolic and catabolic imbalance by activating FoxO1-mediated autophagy in human chondrocytes. FEBS J. 287 (1), 94–107. 10.1111/febs.15009 31330080

[B29] Jellinck P. H. Makin H. L. Sepkovic D. W. Bradlow H. L. (1993). Influence of indole carbinols and growth hormone on the metabolism of 4-androstenedione by rat liver microsomes. J. Steroid Biochem. Mol. Biol. 46 (6), 791–798. 10.1016/0960-0760(93)90320-v 8274413

[B30] Kapoor M. Martel-Pelletier J. Lajeunesse D. Pelletier J. P. Fahmi H. (2011). Role of proinflammatory cytokines in the pathophysiology of osteoarthritis. Nat. Rev. Rheumatol. 7 (1), 33–42. 10.1038/nrrheum.2010.196 21119608

[B31] Khan A. Wang C. Sun X. Killpartrick A. Guo M. (2019). Physicochemical and microstructural properties of polymerized whey protein encapsulated 3, 3'-diindolylmethane nanoparticles. Molecules 24 (4), E702. 10.3390/molecules24040702 PMC6412796 30781356

[B32] Kim H. T. Lo M. Y. Pillarisetty R. (2002). Chondrocyte apoptosis following intraarticular fracture in humans. Osteoarthr. Cartil. 10 (9), 747–749. 10.1053/joca.2002.0828 12202127

[B33] Kim S. J. Lee J. S. Kim S. M. (2012). 3, 3'-Diindolylmethane suppresses growth of human esophageal squamous cancer cells by G1 cell cycle arrest. Oncol. Rep. 27 (5), 1669–1673. 10.3892/or.2012.1662 22293900

[B34] Krenn V. Morawietz L. Burmester G. R. Kinne R. W. Mueller-Ladner U. Muller B. (2006). Synovitis score: Discrimination between chronic low-grade and high-grade synovitis. Histopathology 49 (4), 358–364. 10.1111/j.1365-2559.2006.02508.x 16978198

[B35] Krenn V. Morawietz L. Häupl T. Neidel J. Petersen I. König A. (2002). Grading of chronic synovitis--a histopathological grading system for molecular and diagnostic pathology. Pathol. Res. Pract. 198 (5), 317–325. 10.1078/0344-0338-5710261 12092767

[B36] Laiakis E. C. McCart E. A. Deziel A. Rittase W. B. Bouten R. M. Jha J. (2020). Effect of 3, 3'-diindolylmethane on pulmonary injury following thoracic irradiation in CBA mice. Health Phys. 119 (6), 746–757. 10.1097/hp.0000000000001257 32384373 PMC8579862

[B37] Lee B. D. Yoo J. M. Baek S. Y. Li F. Y. Sok D. E. Kim M. R. (2019). 3, 3'-diindolylmethane promotes BDNF and antioxidant enzyme formation via TrkB/akt pathway activation for neuroprotection against oxidative stress-induced apoptosis in hippocampal neuronal cells. Antioxidants (Basel) 9 (1), E3. 10.3390/antiox9010003 PMC7023184 31861353

[B38] Levine B. Kroemer G. (2008). Autophagy in the pathogenesis of disease. Cell 132 (1), 27–42. 10.1016/j.cell.2007.12.018 18191218 PMC2696814

[B39] Li H. Xie S. Li H. Zhang R. Zhang H. (2020). LncRNA MALAT1 mediates proliferation of LPS treated-articular chondrocytes by targeting the miR-146a-PI3K/Akt/mTOR axis. Life Sci. 254, 116801. 10.1016/j.lfs.2019.116801 31472145

[B40] Li Y. S. Zhang F. J. Zeng C. Luo W. Xiao W. F. Gao S. G. (2016). Autophagy in osteoarthritis. Jt. Bone Spine 83 (2), 143–148. 10.1016/j.jbspin.2015.06.009 26453105

[B41] Li Y. Wu Y. Jiang K. Han W. Zhang J. Xie L. (2019). Mangiferin prevents TBHP-induced apoptosis and ECM degradation in mouse osteoarthritic chondrocytes via restoring autophagy and ameliorates murine osteoarthritis. Oxid. Med. Cell. Longev. 2019, 8783197. 10.1155/2019/8783197 31885823 PMC6815628

[B42] Lian W. S. Ko J. Y. Wu R. W. Sun Y. C. Chen Y. S. Wu S. L. (2018). MicroRNA-128a represses chondrocyte autophagy and exacerbates knee osteoarthritis by disrupting Atg12. Cell Death Dis. 9 (9), 919. 10.1038/s41419-018-0994-y 30206206 PMC6134128

[B43] Lin Z. Miao J. Zhang T. He M. Wang Z. Feng X. (2021). JUNB-FBXO21-ERK axis promotes cartilage degeneration in osteoarthritis by inhibiting autophagy. Aging Cell 20 (2), e13306. 10.1111/acel.13306 33450132 PMC7884044

[B44] Loeser R. F. (2011). Aging and osteoarthritis. Curr. Opin. Rheumatol. 23 (5), 492–496. 10.1097/BOR.0b013e3283494005 21709557 PMC3377970

[B45] Lorenz W. Buhrmann C. Mobasheri A. Lueders C. Shakibaei M. (2013). Bacterial lipopolysaccharides form procollagen-endotoxin complexes that trigger cartilage inflammation and degeneration: Implications for the development of rheumatoid arthritis. Arthritis Res. Ther. 15 (5), R111. 10.1186/ar4291 24020912 PMC3978890

[B46] Luo Q. Yang A. Cao Q. Guan H. (2018). 3, 3'-Diindolylmethane protects cardiomyocytes from LPS-induced inflammatory response and apoptosis. BMC Pharmacol. Toxicol. 19 (1), 71. 10.1186/s40360-018-0262-x 30413180 PMC6230279

[B47] Malemud C. J. (2015). The PI3K/akt/PTEN/mTOR pathway: A fruitful target for inducing cell death in rheumatoid arthritis? Future Med. Chem. 7 (9), 1137–1147. 10.4155/fmc.15.55 26132523

[B48] Martinotti C. Ruiz-Perez L. Deplazes E. Mancera R. L. (2020). Molecular dynamics simulation of small molecules interacting with biological membranes. Chemphyschem 21 (14), 1486–1514. 10.1002/cphc.202000219 32452115

[B49] Maruthanila V. L. Poornima J. Mirunalini S. (2014). Attenuation of carcinogenesis and the mechanism underlying by the influence of indole-3-carbinol and its metabolite 3, 3'-diindolylmethane: A therapeutic marvel. Adv. Pharmacol. Sci. 2014, 832161. 10.1155/2014/832161 24982671 PMC4060499

[B50] Maudens P. Jordan O. Allémann E. (2018). Recent advances in intra-articular drug delivery systems for osteoarthritis therapy. Drug Discov. Today 23 (10), 1761–1775. 10.1016/j.drudis.2018.05.023 29792929

[B51] Mayeux P. R. (1997). Pathobiology of lipopolysaccharide. J. Toxicol. Environ. Health 51 (5), 415–435. 10.1080/00984109708984034 9233377

[B52] Mead T. J. Apte S. S. (2018). ADAMTS proteins in human disorders. Matrix Biol. 71-72, 225–239. 10.1016/j.matbio.2018.06.002 29885460 PMC6146047

[B53] Meng P. Zhang F. Zhang Y. Wei H. Tan S. Guo X. (2020). ADAMTS4 and ADAMTS5 may be considered as new molecular therapeutic targets for cartilage damages with Kashin-Beck Disease. Med. Hypotheses 135, 109440. 10.1016/j.mehy.2019.109440 31734379

[B54] Mizushima N. (2007). Autophagy: Process and function. Genes Dev. 21, 2861–2873. 10.1101/gad.1599207 18006683

[B55] Mizushima N. Levine B. (2020). Autophagy in human diseases. N. Engl. J. Med. 383 (16), 1564–1576. 10.1056/NEJMra2022774 33053285

[B56] Munakarmi S. Chand L. Shin H. B. Jang K. Y. Jeong Y. J. (2020). Indole-3-Carbinol derivative DIM mitigates carbon tetrachloride-induced acute liver injury in mice by inhibiting inflammatory response, apoptosis and regulating oxidative stress. Int. J. Mol. Sci. 21 (6), E2048. 10.3390/ijms21062048 PMC7139345 32192079

[B57] Musumeci G. Aiello F. C. Szychlinska M. A. Di Rosa M. Castrogiovanni P. Mobasheri A. (2015). Osteoarthritis in the XXIst century: Risk factors and behaviours that influence disease onset and progression. Int. J. Mol. Sci. 16 (3), 6093–6112. 10.3390/ijms16036093 25785564 PMC4394521

[B58] Musumeci G. Loreto C. Carnazza M. L. Martinez G. (2011a). Characterization of apoptosis in articular cartilage derived from the knee joints of patients with osteoarthritis. Knee Surg. Sports Traumatol. Arthrosc. 19 (2), 307–313. 10.1007/s00167-010-1215-0 20644910

[B59] Musumeci G. Loreto C. Carnazza M. L. Strehin I. Elisseeff J. (2011b). OA cartilage derived chondrocytes encapsulated in poly(ethylene glycol) diacrylate (PEGDA) for the evaluation of cartilage restoration and apoptosis in an *in vitro* model. Histol. Histopathol. 26 (10), 1265–1278. 10.14670/hh-26.1265 21870330

[B60] Niemann A. Baltes J. Elsässer H. P. (2001). Fluorescence properties and staining behavior of monodansylpentane, a structural homologue of the lysosomotropic agent monodansylcadaverine. J. Histochem. Cytochem. 49 (2), 177–185. 10.1177/002215540104900205 11156686

[B61] Niemann A. Takatsuki A. Elsässer H. P. (2000). The lysosomotropic agent monodansylcadaverine also acts as a solvent polarity probe. J. Histochem. Cytochem. 48 (2), 251–258. 10.1177/002215540004800210 10639491

[B62] Ong I. L. H. Amstad E. (2019). Selectively permeable double emulsions. Small 15 (44), e1903054. 10.1002/smll.201903054 31517446

[B63] Palomera-Sanchez Z. Watson G. W. Wong C. P. Beaver L. M. Williams D. E. Dashwood R. H. (2017). The phytochemical 3, 3'-diindolylmethane decreases expression of AR-controlled DNA damage repair genes through repressive chromatin modifications and is associated with DNA damage in prostate cancer cells. J. Nutr. Biochem. 47, 113–119. 10.1016/j.jnutbio.2017.05.005 28582660 PMC5583029

[B64] Polewska J. (2012). Autophagy--molecular mechanism, apoptosis and cancer. Postepy Hig. Med. Dosw. 66, 921–936. 10.5604/17322693.1021109 23175348

[B65] Qin N. Wei L. Li W. Yang W. Cai L. Qian Z. (2017). Local intra-articular injection of resveratrol delays cartilage degeneration in C57BL/6 mice by inducing autophagy via AMPK/mTOR pathway. J. Pharmacol. Sci. 134 (3), 166–174. 10.1016/j.jphs.2017.06.002 28669597

[B66] Ravikumar B. Sarkar S. Davies J. E. Futter M. Garcia-Arencibia M. Green-Thompson Z. W. (2010). Regulation of mammalian autophagy in physiology and pathophysiology. Physiol. Rev. 90 (4), 1383–1435. 10.1152/physrev.00030.2009 20959619

[B67] Rowan A. D. Litherland G. J. Hui W. Milner J. M. (2008). Metalloproteases as potential therapeutic targets in arthritis treatment. Expert Opin. Ther. Targets 12 (1), 1–18. 10.1517/14728222.12.1.1 18076366

[B68] Ryter S. W. CloonanSuzanne M. Choi Augustine M. K. (2013). Autophagy: A critical regulator of cellular metabolism and homeostasis. Mol. Cells 36 (1), 7–16. 10.1007/s10059-013-0140-8 23708729 PMC3887921

[B69] Rzemieniec J. Litwa E. Wnuk A. Lason W. Krzeptowski W. Kajta M. (2016). Selective aryl hydrocarbon receptor modulator 3, 3'-diindolylmethane impairs AhR and ARNT signaling and protects mouse neuronal cells against hypoxia. Mol. Neurobiol. 53 (8), 5591–5606. 10.1007/s12035-015-9471-0 26476840

[B70] Sasaki H. Takayama K. Matsushita T. Ishida K. Kubo S. Matsumoto T. (2012). Autophagy modulates osteoarthritis-related gene expression in human chondrocytes. Arthritis Rheum. 64 (6), 1920–1928. 10.1002/art.34323 22147463

[B71] Shi H. Xu X. Zhang B. Xu J. Pan Z. Gong A. (2017). 3, 3'-Diindolylmethane stimulates exosomal Wnt11 autocrine signaling in human umbilical cord mesenchymal stem cells to enhance wound healing. Theranostics 7 (6), 1674–1688. 10.7150/thno.18082 28529644 PMC5436520

[B72] Sukhikh S. Noskova S. Ivanova S. Ulrikh E. Izgaryshev A. Babich O. (2021). Chondroprotection and molecular mechanism of action of phytonutraceuticals on osteoarthritis. Molecules 26 (8), 2391. 10.3390/molecules26082391 33924083 PMC8074261

[B73] Takahata Y. Nakamura E. Hata K. Wakabayashi M. Murakami T. Wakamori K. (2019). Sox4 is involved in osteoarthritic cartilage deterioration through induction of ADAMTS4 and ADAMTS5. Faseb J. 33 (1), 619–630. 10.1096/fj.201800259R 30016600

[B74] Takayama K. Kawakami Y. Kobayashi M. Greco N. Cummins J. H. Matsushita T. (2014). Local intra-articular injection of rapamycin delays articular cartilage degeneration in a murine model of osteoarthritis. Arthritis Res. Ther. 16 (6), 482. 10.1186/s13075-014-0482-4 25403236 PMC4269094

[B75] Vaishya R. Pariyo G. B. Agarwal A. K. Vijay V. (2016). Non-operative management of osteoarthritis of the knee joint. J. Clin. Orthop. Trauma 7 (3), 170–176. 10.1016/j.jcot.2016.05.005 27489412 PMC4949406

[B76] Vázquez C. L. Colombo M. I. (2009). Assays to assess autophagy induction and fusion of autophagic vacuoles with a degradative compartment, using monodansylcadaverine (MDC) and DQ-BSA. Methods Enzymol. 452, 85–95. 10.1016/s0076-6879(08)03606-9 19200877

[B77] Verlaan L. Boekesteijn R. J. Oomen P. W. Liu W. Y. Peters M. J. M. Witlox M. A. (2018). Biomechanical alterations during sit-to-stand transfer are caused by a synergy between knee osteoarthritis and obesity. Biomed. Res. Int. 2018, 3519498. 10.1155/2018/3519498 30627551 PMC6304591

[B78] Vincenti M. P. Brinckerhoff C. E. (2002). Transcriptional regulation of collagenase (MMP-1, MMP-13) genes in arthritis: Integration of complex signaling pathways for the recruitment of gene-specific transcription factors. Arthritis Res. 4 (3), 157–164. 10.1186/ar401 12010565 PMC128926

[B79] Wang A. Fang S. Zhong L. Lu M. Zhou H. Huang W. (2022). Shikonin, a promising therapeutic drug for osteoarthritis that acts via autophagy activation. Int. Immunopharmacol. 106, 108563. 10.1016/j.intimp.2022.108563 35176588

[B80] Weng J. R. Bai L. Y. Chiu C. F. Wang Y. C. Tsai M. H. (2012). The dietary phytochemical 3, 3'-diindolylmethane induces G2/M arrest and apoptosis in oral squamous cell carcinoma by modulating Akt-NF-κB, MAPK, and p53 signaling. Chem. Biol. Interact. 195 (3), 224–230. 10.1016/j.cbi.2012.01.003 22290291

[B81] Woolf A. D. Pfleger B. (2003). Burden of major musculoskeletal conditions. Bull. World Health Organ. 81 (9), 646–656. 10.1590/S0042-96862003000900007 14710506 PMC2572542

[B82] Wu G. J. Chen T. G. Chang H. C. Chiu W. T. Chang C. C. Chen R. M. (2007). Nitric oxide from both exogenous and endogenous sources activates mitochondria-dependent events and induces insults to human chondrocytes. J. Cell. Biochem. 101 (6), 1520–1531. 10.1002/jcb.21268 17492650

[B83] Xu K. He Y. Moqbel S. A. A. Zhou X. Wu L. Bao J. (2021). SIRT3 ameliorates osteoarthritis via regulating chondrocyte autophagy and apoptosis through the PI3K/Akt/mTOR pathway. Int. J. Biol. Macromol. 175, 351–360. 10.1016/j.ijbiomac.2021.02.029 33556400

[B84] Xue J. F. Shi Z. M. Zou J. Li X. L. (2017). Inhibition of PI3K/AKT/mTOR signaling pathway promotes autophagy of articular chondrocytes and attenuates inflammatory response in rats with osteoarthritis. Biomed. Pharmacother. 89, 1252–1261. 10.1016/j.biopha.2017.01.130 28320092

[B85] Ye Y. Fang Y. Xu W. Wang Q. Zhou J. Lu R. (2016). 3, 3'-Diindolylmethane induces anti-human gastric cancer cells by the miR-30e-ATG5 modulating autophagy. Biochem. Pharmacol. 115, 77–84. 10.1016/j.bcp.2016.06.018 27372603

[B86] Ye Y. Li X. Wang Z. Ye F. Xu W. Lu R. (2021). 3, 3'-Diindolylmethane induces gastric cancer cells death via STIM1 mediated store-operated calcium entry. Int. J. Biol. Sci. 17 (5), 1217–1233. 10.7150/ijbs.56833 33867841 PMC8040462

[B87] Yoshino S. Sasatomi E. Ohsawa M. (2000). Bacterial lipopolysaccharide acts as an adjuvant to induce autoimmune arthritis in mice. Immunology 99 (4), 607–614. 10.1046/j.1365-2567.2000.00015.x 10792509 PMC2327198

[B88] Zamli Z. Sharif M. (2011). Chondrocyte apoptosis: A cause or consequence of osteoarthritis? Int. J. Rheum. Dis. 14 (2), 159–166. 10.1111/j.1756-185X.2011.01618.x 21518315

[B89] Zeng R. Lu X. Lin J. Ron Z. Fang J. Liu Z. (2021). FOXM1 activates JAK1/STAT3 pathway in human osteoarthritis cartilage cell inflammatory reaction. Exp. Biol. Med. 246 (6), 644–653. 10.1177/1535370220974933 PMC7988721 33297736

[B90] Zhang Y. Vasheghani F. Li Y. H. Blati M. Simeone K. Fahmi H. (2015). Cartilage-specific deletion of mTOR upregulates autophagy and protects mice from osteoarthritis. Ann. Rheum. Dis. 74 (7), 1432–1440. 10.1136/annrheumdis-2013-204599 24651621

[B91] Zhao Z. Li Y. Wang M. Zhao S. Zhao Z. Fang J. (2020). Mechanotransduction pathways in the regulation of cartilage chondrocyte homoeostasis. J. Cell. Mol. Med. 24 (10), 5408–5419. 10.1111/jcmm.15204 32237113 PMC7214151

