# Supplementary material for: 3,3′-diindolylmethane inhibits LPS-induced human chondrocytes apoptosis and extracellular matrix degradation by activating PI3K-Akt-mTOR-mediated autophagy
Source: Front Pharmacol. 2022 Nov 10;13:999851. doi: 10.3389/fphar.2022.999851 (PMC9684728; doi:10.3389/fphar.2022.999851)
Supplement: Supplementary file 1 [file Table1.DOCX]

Supplementary Material

**Table S1.** The primer sequences used for qRT-PCR

| **Gene** |  | **Primer Sequence (5′-3′)** |
| --- | --- | --- |
| Collagen II | F | 5′-GATGCCACACTCAAGTCCCTCA-3′ |
|  | R | 5′-TGCTGCTCCACCAGTTCTTCTT-3′ |
| Aggrecan | F | 5′-TCACCACCGAGCCAGAAAAC-3′ |
|  | R | 5′-GCTCTTCCGAGGCTGATGG-3′ |
| ADAMTS-5 | F | 5′-GAGCCTGGAAGTGAGCAAGAA-3′ |
|  | R | 5′-CACATAAATCCTCCCGAGTAAACA-3′ |
| MMP-13 | F | 5′-AGACCCCAACCCTAAACATCC-3′ |
|  | R | 5′-AAAACAGCTCCGCATCAACC-3′ |
| GAPDH | F | 5′-GGAAGCTTGTCATCAATGGAAATC-3′ |
|  | R | 5′-TGATGACCCTTTTGGCTCCC-3′ |
