# Supplementary figures and images for: 3,3′-diindolylmethane inhibits LPS-induced human chondrocytes apoptosis and extracellular matrix degradation by activating PI3K-Akt-mTOR-mediated autophagy
Source: Front Pharmacol. 2022 Nov 10;13:999851. doi: 10.3389/fphar.2022.999851 (PMC9684728; doi:10.3389/fphar.2022.999851)

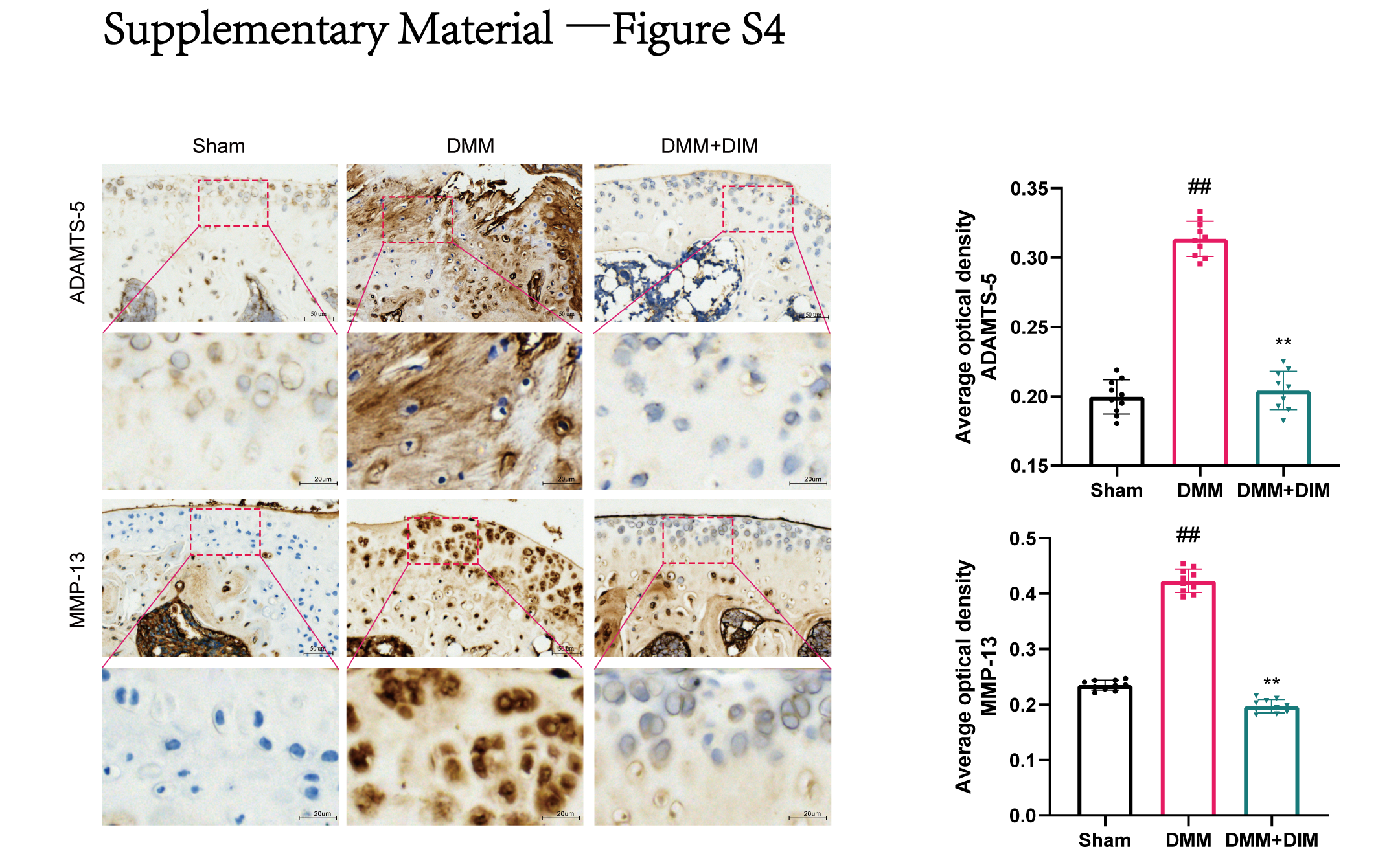

Supplement: Supplementary file 2 [file Image3.TIF]

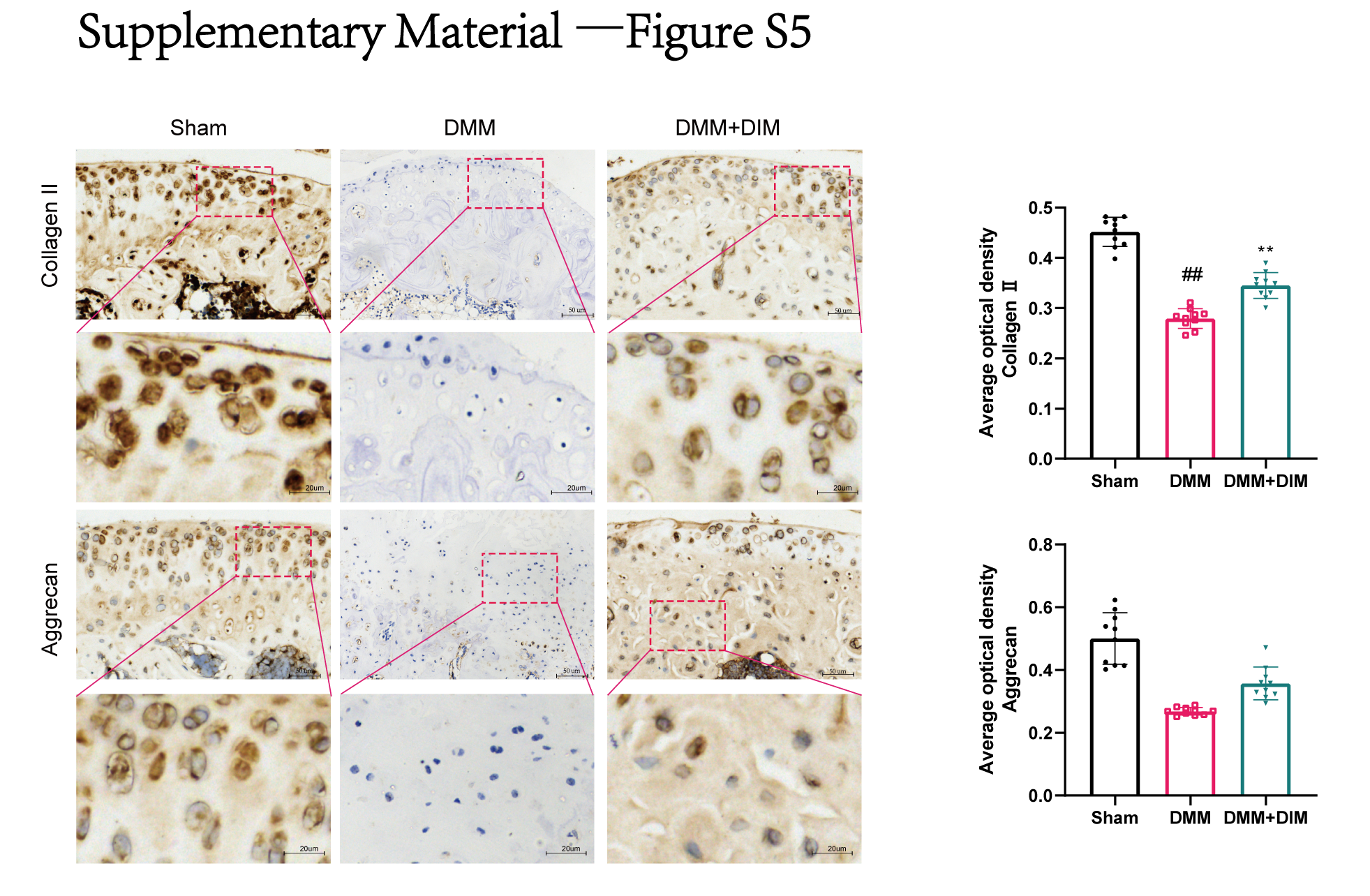

Supplement: Supplementary file 3 [file Image4.TIF]

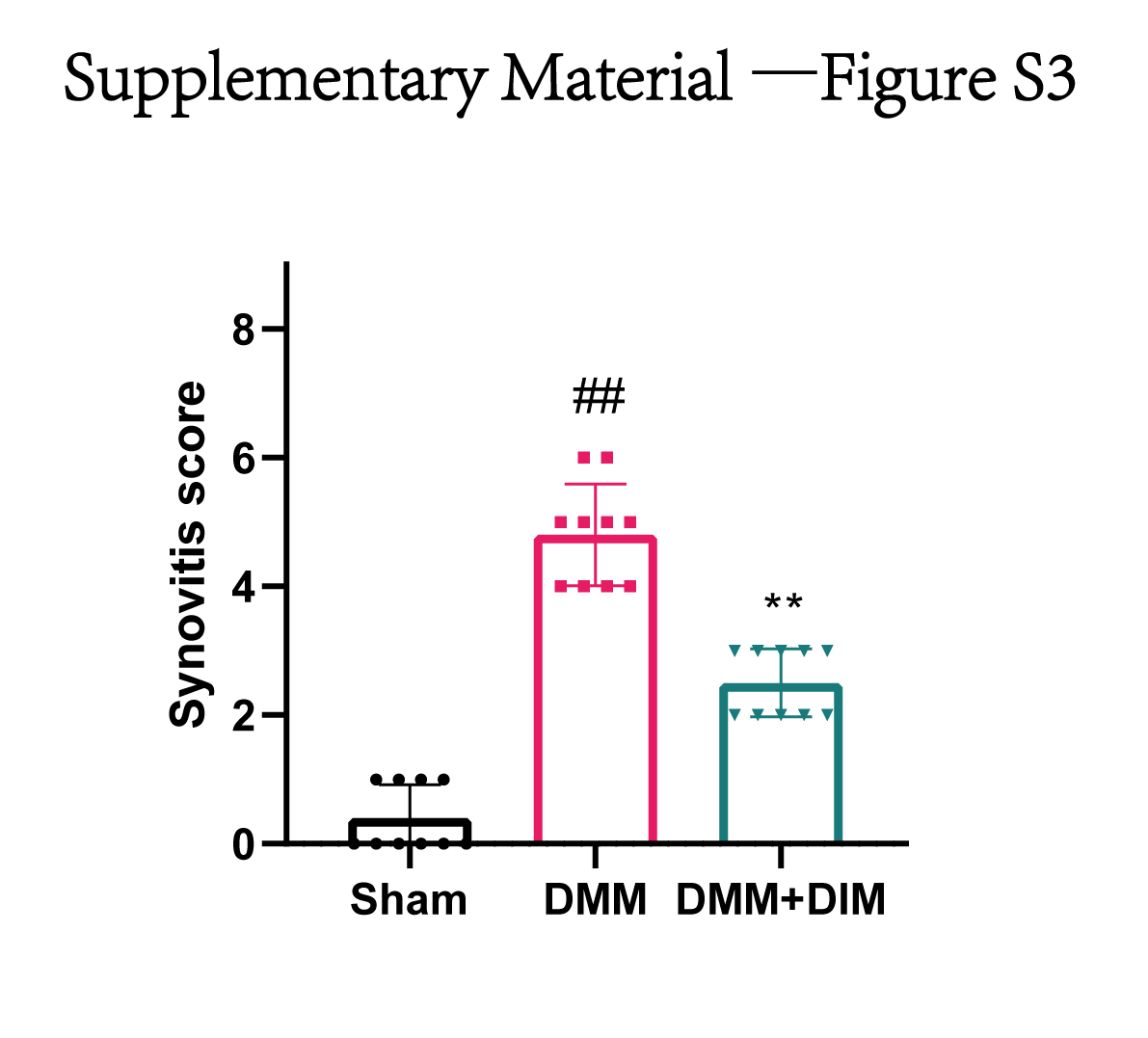

Supplement: Supplementary file 4 [file Image2.TIF]

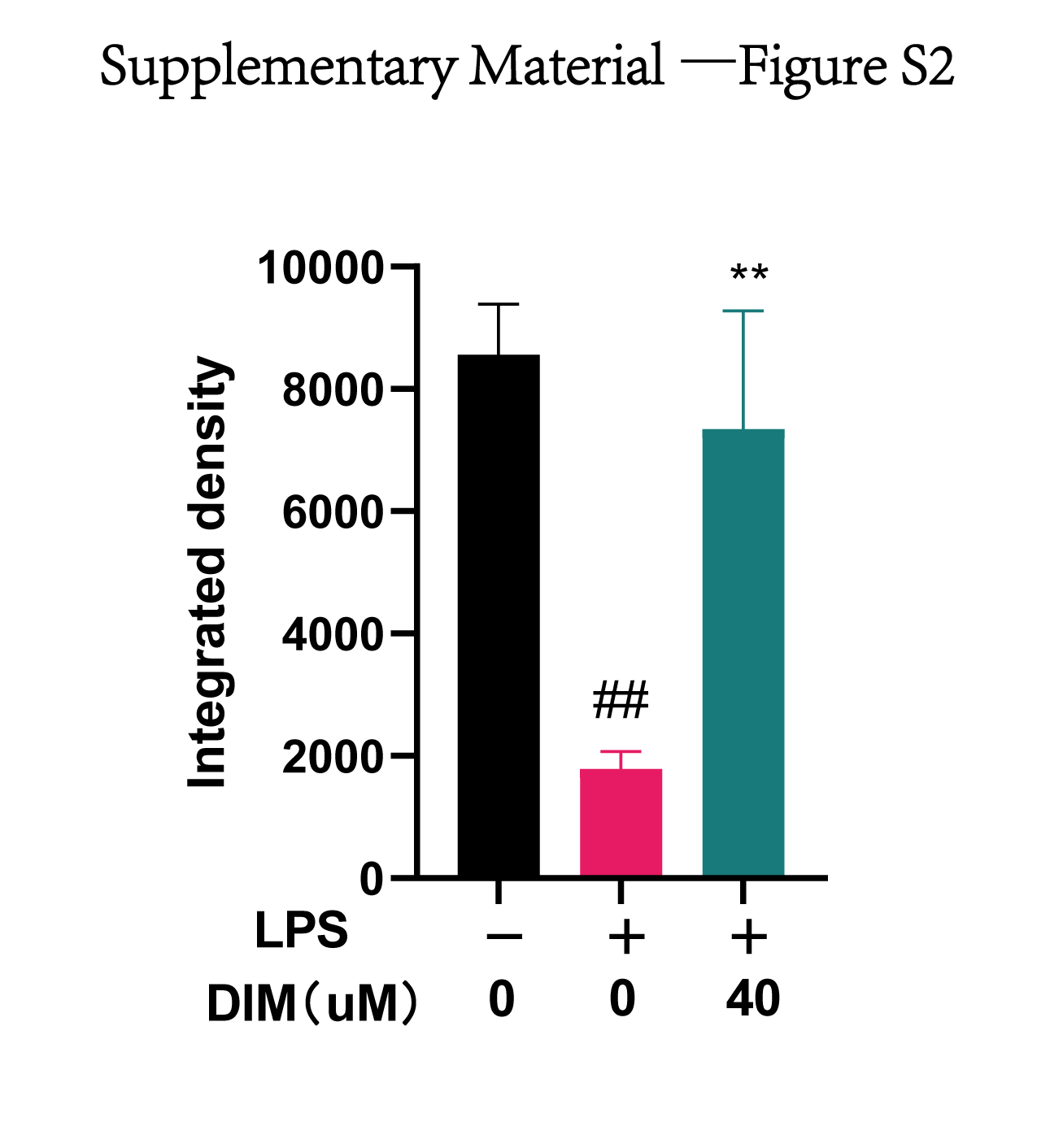

Supplement: Supplementary file 5 [file Image1.TIF]
