# Supplementary material for: 3,3′-diindolylmethane inhibits LPS-induced human chondrocytes apoptosis and extracellular matrix degradation by activating PI3K-Akt-mTOR-mediated autophagy
Source: Front Pharmacol. 2022 Nov 10;13:999851. doi: 10.3389/fphar.2022.999851 (PMC9684728; doi:10.3389/fphar.2022.999851)
Supplement: Supplementary file 6 [file Table2.docx]

Supplementary Material- Figure Legend of S2-S5

**FIGURE S2. The quantitative statistical analysis of GAGs expression**

**(S2)** The integrated density was measured with the ImageJ software (U.S. National Institutes of Health, Bethesda). The data are presented as mean ± SD values from three independent experiments. Using one-way ANOVA, NS, no statistical difference. ^#^ *p* < 0.05 vs. the control; ^# #^ *p* < 0.01 vs. the control; * *p* < 0.05 vs. the LPS group; ** *p* < 0.01 vs. the LPS group.

**FIGURE S3. Mice knee joint synovitis scores**

**(S3)** Mice knee joint synovitis scores. The data are presented as mean ± SD values from three independent experiments (n = 10 in each group). Using one-way ANOVA, NS, no statistical difference; ^#^ *p* < 0.05 vs. the Sham group; ^##^ *p* < 0.01, vs. the Sham group, * *p* < 0.05 vs. the DMM group; ** *p* < 0.01 vs. the DMM group.

**FIGURE S4. Immunohistochemical staining of ADAMTS-5, MMP-13 expressions in Mice articular cartilage of different experimental groups**

**(S4)** Immunohistochemical staining of ADAMTS-5, MMP-13 expressions in cartilage samples of different experimental groups (scale bar, 50 µm; partially enlarged image scale bar, 20 µm) and AODs were analyzed by the ImageJ software (U.S. National Institutes of Health, Bethesda). The data are presented as mean ± SD values from three independent experiments (n = 10 in each group). Using one-way ANOVA, NS, no statistical difference; ^#^ *p* < 0.05 vs. the Sham group; ^##^ *p* < 0.01, vs. the Sham group, * *p* < 0.05 vs. the DMM group; ** *p* < 0.01 vs. the DMM group.

**FIGURE S5. Immunohistochemical staining of Collagen II, Aggrecan expressions in Mice articular cartilage of different experimental groups**

**(S5)** Immunohistochemical staining of Collagen II, Aggrecan expressions in cartilage samples of different experimental groups (scale bar, 50 µm; partially enlarged image scale bar, 20 µm) and AODs were analyzed by the ImageJ software (U.S. National Institutes of Health, Bethesda). The data are presented as mean ± SD values from three independent experiments (n = 10 in each group). Using one-way ANOVA, NS, no statistical difference; ^#^ *p* < 0.05 vs. the Sham group; ^##^ *p* < 0.01, vs. the Sham group, * *p* < 0.05 vs. the DMM group; ** *p* < 0.01 vs. the DMM group.
